# A systematic review of healthcare provider-targeted mobile applications for non-communicable diseases in low- and middle-income countries

**DOI:** 10.1038/s41746-022-00644-3

**Published:** 2022-07-19

**Authors:** Pascal Geldsetzer, Sergio Flores, Grace Wang, Blanca Flores, Abu Bakarr Rogers, Aditi Bunker, Andrew Y. Chang, Rebecca Tisdale

**Affiliations:** 1 Division of Primary Care and Population Health, Department of Medicine, Stanford University, Stanford, CA USA; 2 Heidelberg Institute of Global Health, Heidelberg University, Heidelberg, Germany; 3 Center for Innovation in Global Health, Stanford University, Stanford, CA USA; 4 Department of Epidemiology and Global Health, Umeå University, Umeå, Sweden; 5 Department of Biology, Stanford University, Stanford, CA USA; 6 Heidelberg University Hospital, Heidelberg, Germany; 7 Stanford University School of Medicine, Stanford, CA USA; 8 Department of Epidemiology and Population Health, Stanford University, Stanford, CA USA; 9 Department of Medicine, Stanford University School of Medicine, Stanford, CA USA; 10 Stanford Cardiovascular Institute, Stanford University, Stanford, CA USA; 11 Veterans Affairs Palo Alto Healthcare System, Center for Innovation to Implementation, Menlo Park, CA USA; 12 Department of Health Policy, School of Medicine, and Center for Health Policy, Freeman Spogli Institute for International Studies, Stanford University, Stanford, CA USA

**Keywords:** Disease prevention, Health policy

## Abstract

Mobile health (mHealth) interventions hold promise for addressing the epidemic of noncommunicable diseases (NCDs) in low- and middle-income countries (LMICs) by assisting healthcare providers managing these disorders in low-resource settings. We aimed to systematically identify and assess provider-facing mHealth applications used to screen for, diagnose, or monitor NCDs in LMICs. In this systematic review, we searched the indexing databases of PubMed, Web of Science, and Cochrane Central for studies published between January 2007 and October 2019. We included studies of technologies that were: (i) mobile phone- or tablet-based, (ii) able to screen for, diagnose, or monitor an NCD of public health importance in LMICs, and (iii) targeting health professionals as users. We extracted disease type, intervention purpose, target population, study population, sample size, study methodology, technology stage, country of development, operating system, and cost. Our initial search retrieved 13,262 studies, 315 of which met inclusion criteria and were analyzed. Cardiology was the most common clinical domain of the technologies evaluated, with 89 publications. mHealth innovations were predominantly developed using Apple’s iOS operating system. Cost data were provided in only 50 studies, but most technologies for which this information was available cost less than 20 USD. Only 24 innovations targeted the ten NCDs responsible for the greatest number of disability-adjusted life years lost globally. Most publications evaluated products created in high-income countries. Reported mHealth technologies are well-developed, but their implementation in LMICs faces operating system incompatibility and a relative neglect of NCDs causing the greatest disease burden.

## Introduction

The rapid rise of noncommunicable disease (NCD) prevalence in low- and middle-income countries (LMICs, as defined by the World Bank 2021 country classifications)^1^ has become a critical public health challenge, triggered by multiple global demographic trends such as population aging, economic development, and dietary/lifestyle transitions^2–5^. In 2019, NCDs, which include major chronic diseases such as hypertension, diabetes, depression, and their sequelae, were responsible for an estimated 42 million deaths globally, 77% of which were in LMICs^6^. People living in poor nations are particularly susceptible to NCDs due to health system vulnerabilities and high socioeconomic inequality^7–9^. In particular, healthcare provider shortages in low-resource settings exacerbate these challenges, fueling the urgent need for innovative solutions in these locations^10,11^.LMIC

One potential solution to stemming the stresses that NCDs place upon healthcare systems has been the incorporation of mHealth (mobile health) technologies^10,12–14^. By standardizing complex protocols and adapting diagnostic and monitoring equipment for simplified use in ubiquitous mobile devices such as cellular telephones and tablets, the efficiency and practice range of physicians and nurses can be extended, while other tasks can be shifted to community health workers (CHWs). Indeed, over the past several decades, there has been an unprecedented increase in the number of mobile phone and Internet users in LMICs, owing to a steep decline in the price of these devices and their connectivity services^15^. As of 2021, there were an estimated 5.27 billion unique mobile-phone users worldwide and 4.72 billion Internet users^16^; LMICs account for 2.9 billon of these users^17^, with cell phone and mobile internet connectivity penetration in these countries over 90% and around 40%, respectively^18,19^. It is no surprise that CHWs are increasingly being equipped with smartphones and tablets, as many mHealth applications represent focused, protocolized programs that are ideal for CHW use^20,21^.

Despite these advantages, challenges such as limited internet connectivity, lack of technical support, disparity in clinical resources between technology development and usage settings, and insufficient training of users can limit the use and expansion of mHealth in resource-constrained regions^22,23^. As such, maximizing the benefits of mobile technologies for healthcare workers in LMICs will require healthcare professionals, program managers, researchers, and policymakers to understand the context and limitations of these tools. To our knowledge, there is currently no up-to-date, comprehensive systematic overview of mHealth technologies for NCD management targeted for healthcare provider use in LMIC settings. This systematic review thus aims to identify and summarize existing provider-facing mobile-phone and tablet-based applications that can be used to screen for, diagnose, or monitor NCDs in LMICs.

## Results

**Fig. 1:**
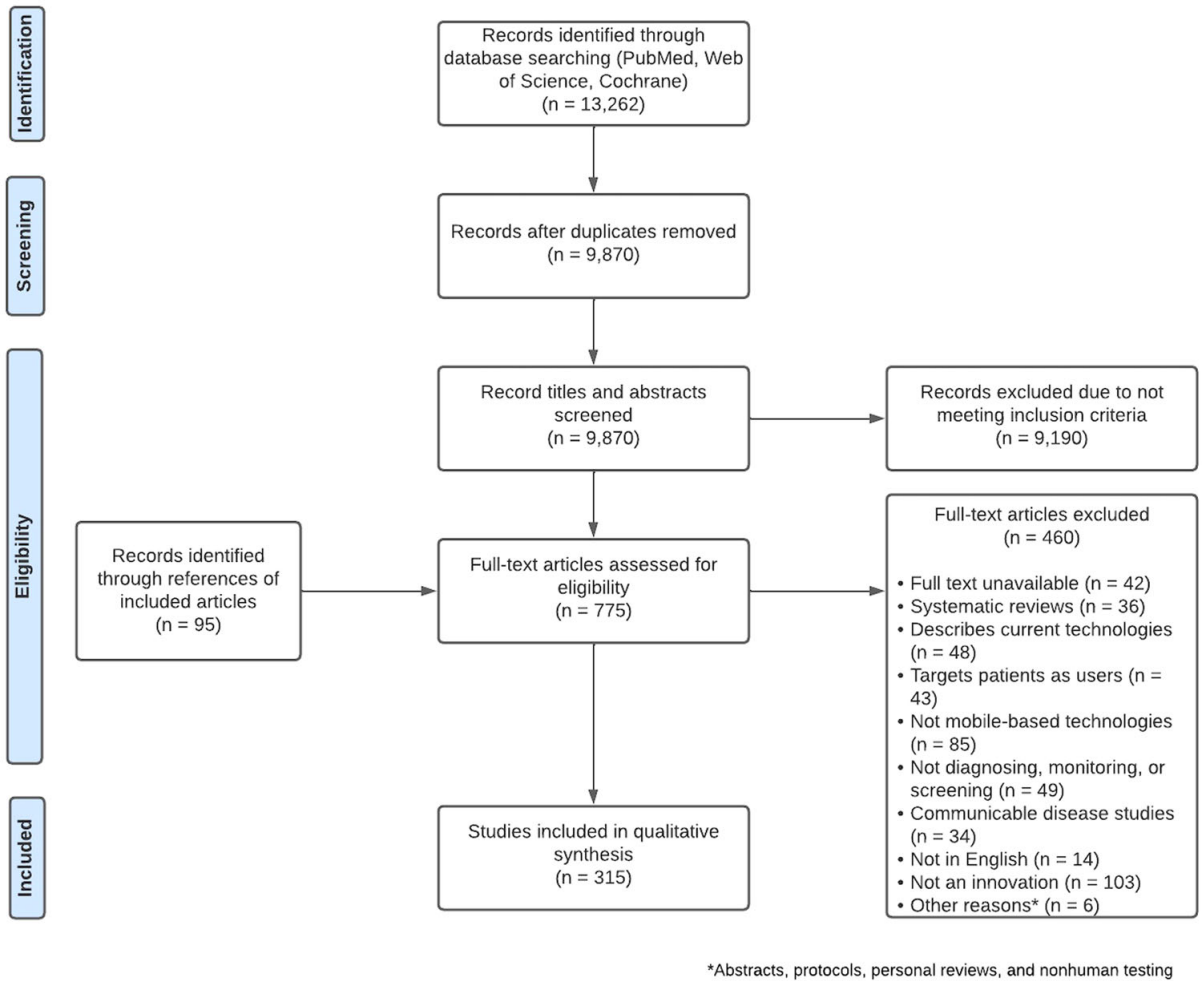
PRISMA diagram.

Our initial search of all databases retrieved 13,262 results. After duplicates were removed, abstracts screened, full texts reviewed, and articles identified from reference lists of included publications were added, 315 studies met our inclusion criteria (Fig. 1).

Articles were excluded if they described or evaluated: (i) interventions not meant for diagnosis, screening and/or monitoring (*n* = 49); *ii)* non-mobile technology-based interventions (*n* = 85); (iii) interventions targeting patients instead of health professionals as users (*n* = 43); (iv) the general status of current technologies (*n* = 48); (v) communicable diseases (*n* = 34), or that *vi)* did not have a full text available (*n* = 42); (vii) were not available in English (*n* = 14); (viii) were systematic reviews (*n* = 36); (ix) were study protocols or involved non-human testing (*n* = 6); (x) presented technology that merely digitalized protocols, scores or other procedures that could be done on paper (*n* = 103). A detailed overview of our results is presented in Table 1.

**Table 1:** Summary of characteristics for noncommunicable disease studies by clinical specialty.

		Total^a^ (*n* = 315)	Cardiology (*n* = 89)	Ophthalmology and Otorhinolaryngology (*n* = 51)	Neurology (*n* = 38)	General Medicine (*n* = 22)	Hematology (*n* = 18)	Maternal and Child Healthcare (*n* = 17)	Oncology (*n* = 15)	Dermatology (*n* = 12)	Endocrinology (*n* = 11)	Nutrition and Sports Medicine (*n* = 8)	Psychiatry (*n* = 7)	Orthopedics and Traumatology (*n* = 6)	Surgery and anesthesiology (*n* = 6)	Nephrology and urology (*n* = 5)	Pulmonary Medicine (*n* = 4)	Rheumatology (*n* = 4)	Allergology and Immunology (*n* = 2)
Year of publication	Total	**315**	**89**	**51**	**38**	**22**	**18**	**17**	**15**	**12**	**11**	**8**	**7**	**6**	**6**	**5**	**4**	**4**	**2**
	2006–2008	**5 (1.6)**	2 (2.2)	–	–	2 (9.1)	–	–	–	1 (8.3)	–	–	–	–	–	–	–	–	–
	2009–2011	**38 (12.0)**	8 (9.0)	1 (2.0)	5 (12.8)	6 (27.3)	2 (11.1)	1 (5.9)	3 (20.0)	3 (25.0)	3 (27.3)	1 (12.5)	2 (28.6)	1 (16.7)	–	–	1 (25.0)	1 (25.0)	–
	2012–2014	**72 (22.8)**	18 (20.2)	13 (25.5)	10 (25.6)	3 (13.6)	5 (27.8)	2 (11.8)	1 (6.7)	5 (41.7)	3 (27.3)	3 (37.5)	2 (28.6)	3 (50.0)	2 (33.3)	1 (20.0)	–	–	1 (50.0)
	2015–2017	**117 (37.0)**	34 (38.2)	22 (43.1)	13 (33.3)	9 (40.9)	7 (38.9)	7 (41.2)	7 (46.7)	1 (8.3)	1 (9.1)	4 (50.0)	3 (42.9)	1 (16.7)	4 (66.7)	1 (20.0)	–	2 (50.0)	1 (50.0)
	2018–2020	**83 (26.6)**	27 (30.3)	15 (29.4)	10 (25.6)	2 (9.1)	4 (22.2)	7 (41.2)	4 (26.7)	2 (16.7)	4 (36.4)	–	–	1 (16.7)	–	3 (60.0)	3 (75.0)	1 (25.0)	–
Author affiliation	Total	**315**	**89**	**51**	**38**	**22**	**18**	**17**	**15**	**12**	**11**	**8**	**7**	**6**	**6**	**5**	**4**	**4**	**2**
	North America	**117 (37.3)**	33 (37.1)	14 (27.5)	14 (38.5)	7 (31.8)	12 (66.7)	4 (23.5)	9 (60.0)	3 (25.0)	3 (27.3)	5 (62.5)	3 (42.9)	2 (33.3)	2 (33.3)	3 (60.0)	1 (25.0)	–	2
	South America	**4 (1.3)**	1 (1.1)	1 (2.0)	–	–	–	1 (5.9)	–	1 (8.3)	–	–	–	–	–	–	–	–	–
	Europe	**60 (19.0)**	16 (18.0)	11 (21.6)	10 (25.6)	5 (22.7)	–	3 (17.6)	1 (6.7)	7 (58.3)	2 (18.2)	–	1 (14.3)	1 (16.7)	2 (33.3)	–	–	1 (25.0)	–
	Africa	**13 (4.1)**	1 (1.1)	7 (13.7)	–	–	–	3 (17.6)	1 (6.7)	–	–	–	–	–	–	–	1 (25.0)	–	–
	Asia	**77 (24.4)**	24 (27.0)	12 (23.5)	10 (25.6)	7 (31.8)	6 (33.3)	2 (11.8)	3 (20.0)	1 (8.3)	3 (27.3)	1 (12.5)	1 (14.3)	2 (33.3)	1 (16.7)	1 (20.0)	–	3 (75.0)	–
	Oceania	**18 (5.7)**	7 (7.9)	2 (3.9)	2 (5.1)	–	–	1 (5.9)	–	–	1 (9.1)	1 (12.5)	1 (14.3)	–	1 (16.7)	1 (20.0)	1 (25.0)	–	–
	Multinational	**26 (8.2)**	7 (7.9)	4 (7.8)	2 (5.1)	3 (13.6)	–	3 (17.6)	1 (6.7)	–	2 (18.2)	1 (12.5)	1 (14.3)	1 (16.7)	–	–	1 (25.0)	–	–
Type of device	Total	**343**	**93**	**59**	**46**	**22**	**19**	**17**	**16**	**13**	**13**	**8**	**7**	**6**	**6**	**5**	**4**	**6**	**2**
	Armband/smartwatch	**12 (3.8)**	9 (10.1)	–	1 (2.6)	1 (4.5)	–	–	–	–	–	–	–	–	–	–	1 (25.0)	–	–
	Smartphones	**252 (80.1)**	71 (79.8)	42 (82.4)	29 (76.9)	14 (63.6)	16 (88.9)	13 (76.5)	12 (80.0)	9 (75.0)	9 (81.8)	8	4 (57.1)	6	6	4 (80.0)	3 (75.0)	4	2
	Mobile phones	**21 (6.6)**	4 (4.5)	1 (2.0)	1 (2.6)	4 (18.2)	–	3 (17.6)	–	3 (25.0)	2 (18.2)	–	2 (28.6)	–	–	1 (20.0)	–	–	–
	Tablets	**31 (9.8)**	4 (4.5)	12 (23.5)	9 (23.1)	–	1 (5.6)	–	1 (6.7)	1 (8.3)	2 (18.2)	–	–	–	–	–	–	1 (25.0)	–
	iPod devices	**10 (3.2)**	1 (1.1)	3 (5.9)	3 (7.7)	1 (4.5)	–	1 (5.9)	1 (6.7)	–	–	–	–	–	–	–	–	–	–
	PC	**5 (1.6)**	1 (1.1)	1 (2.0)	–	–	1 (5.6)	–	1 (6.7)	–	–	–	–	–	–	–	–	1 (25.0)	–
	Other wireless devices	**11 (3.5)**	3 (3.4)	–	3 (7.7)	2 (9.1)	1 (5.6)	–	1 (6.7)	–	–	–	1 (14.3)	–	–	–	–	–	–
Development stage	Total	**315**	**89**	**51**	**38**	**22**	**18**	**17**	**15**	**12**	**11**	**8**	**7**	**6**	**6**	**5**	**4**	**4**	**2**
	Proof of Concept/principle	**20 (6.3)**	6 (6.7)	2 (3.9)	–	3 (13.6)	1 (5.6)	1 (5.9)	3 (20.0)	3 (25.0)	–	–	–	–	–	–	–	1 (25.0)	–
	In development	**6 (2.2)**	1 (1.1)	2 (3.9)	1 (5.1)	–	2 (11.1)	–	–	–	–	–	–	–	–	–	–	–	–
	Prototype	**50 (15.8)**	15 (16.9)	6 (11.8)	6 (15.4)	7 (31.8)	2 (11.1)	3 (17.6)	2 (13.3)	2 (16.7)	1 (9.1)	2 (25.0)	–	–	1 (16.7)	2 (40.0)	1 (25.0)	–	–
	Pilot	**6 (1.9)**	1 (1.1)	–	2 (5.1)	–	–	1 (5.9)	1 (6.7)	–	1 (9.1)	–	–	–	–	–	–	–	–
	Validation trial/test in clinical trial	**6 (1.9)**	2 (2.2)	2 (3.9)	–	1 (4.5)	–	–	–	–	1 (9.1)	–	–	–	–	–	–	–	–
	Available/developed	**211 (66.8)**	60 (67.4)	38 (74.5)	28 (71.8)	9 (40.9)	13 (72.2)	10 (58.8)	8 (53.3)	5 (41.7)	7 (63.6)	6 (75.0)	7	6	4 (66.7)	3 (60.0)	2 (50.0)	3 (75.0)	2
	Not specified	**16 (5.1)**	4 (4.5)	1 (2.0)	1 (2.6)	2 (9.1)	–	2 (11.8)	1 (6.7)	2 (16.7)	1 (9.1)	–	–	–	1 (16.7)	–	1 (25.0)	–	–
Operating System	Total	**315**	**89**	**51**	**38**	**22**	**18**	**17**	**15**	**12**	**11**	**8**	**7**	**6**	**6**	**5**	**4**	**4**	**2**
	iOS	**113 (35.8)**	33 (37.1)	28 (54.9)	18 (46.2)	3 (13.6)	3 (16.7)	5 (29.4)	5 (33.3)	5 (41.7)	2 (18.2)	3 (37.5)	–	5 (83.3)	1 (16.7)	2 (40.0)	–	–	–
	Android	**98 (31.0)**	21 (23.6)	15 (29.4)	11 (28.2)	9 (40.9)	8 (44.4)	5 (29.4)	4 (26.7)	3 (25.0)	7 (63.6)	2 (25.0)	4 (57.1)	1 (16.7)	2 (33.3)	2 (40.0)	–	2 (50.0)	2
	Windows	**5 (1.6)**	4 (4.5)	–	–	–	–	–	1 (6.7)	–	–	–	–	–	–	–	–	–	–
	Blackberry	**2 (0.6)**	–	1 (2.0)	1 (2.6)	–	–	–	–	–	–	–	–	–	–	–	–	–	–
	MultiOS	**21 (6.6)**	8 (9.0)	2 (3.9)	1 (2.6)	3 (13.6)	1 (5.6)	3 (17.6)	1 (6.7)	–	–	1 (12.5)	–	–	1 (16.7)	–	–	–	–
	Others	**3 (0.9)**	2 (2.2)	–	–	1 (4.5)	–	–	–	–	–	–	–	–	–	–	–	–	–
	Not specified	**73 (23.4)**	21 (23.6)	5 (9.8)	7 (20.5)	6 (27.3)	6 (33.3)	4 (23.5)	4 (26.7)	4 (33.3)	2 (18.2)	2 (25.0)	3 (42.9)	–	2 (33.3)	1 (20.0)	4	2 (50.0)	–
Internet required to work	Total	**315**	**89**	**51**	**38**	**22**	**18**	**17**	**15**	**12**	**11**	**8**	**7**	**6**	**6**	**5**	**4**	**4**	**2**
	Yes	**56 (17.7)**	16 (18.0)	6 (11.8)	11 (28.2)	5 (22.7)	2 (11.1)	1 (5.9)	3 (20.0)	2 (16.7)	4 (36.4)	2 (25.0)	1 (14.3)	1 (16.7)	1 (16.7)	–	–	1 (25.0)	–
	No	**258 (81.6)**	72 (80.9)	45 (88.2)	27 (69.2)	17 (77.3)	16 (88.9)	16 (94.1)	12 (80.0)	10 (83.3)	7 (63.6)	6 (75.0)	6 (85.7)	5 (83.3)	5 (83.3)	5	4	3 (75.0)	2
	Not specified	**1 (0.6)**	1 (1.1)	–	–	–	–	–	–	–	–	–	–	–	–	–	–	–	–
Bluetooth required to work	Total	**315**	**89**	**51**	**38**	**22**	**18**	**17**	**15**	**12**	**11**	**8**	**7**	**6**	**6**	**5**	**4**	**4**	**2**
	Yes	**59 (18.7)**	28 (31.5)	1 (2.0)	4 (10.3)	8 (36.4)	2 (11.1)	2 (11.8)	3 (20.0)	1 (8.3)	3 (27.3)	2 (25.0)	2 (28.6)	1 (16.7)	–	–	2 (50.0)	–	–
	No	**255 (80.7)**	60 (67.4)	50 (98.0)	34 (87.2)	14 (63.6)	16 (88.9)	15 (88.2)	12 (80.0)	11 (91.7)	8 (72.7)	6 (75.0)	5 (71.4)	5 (83.3)	6	5	2 (50.0)	4	2
	Not specified	**1 (0.6)**	1 (1.1)	–	–	–	–	–	–	–	–	–	–	–	–	–	–	–	–
Accessories required to work	Total	**315**	**89**	**51**	**38**	**22**	**18**	**17**	**15**	**12**	**11**	**8**	**7**	**6**	**6**	**5**	**4**	**4**	**2**
	Yes	**209 (66.1)**	54 (60.7)	37 (72.5)	19 (50,0)	16 (72.7)	16 (88.9)	10 (58.8)	12 (80.0)	7 (58.3)	8 (72.7)	6 (75.0)	3 ()42.9	3 (50.0)	4 (66.7)	4 (80.0)	4	4	2
	No	**106 (33.9)**	35 (39.3)	14 (27.5)	19 (50,0)	6 (27.3)	2 (11.1)	7 (41.2)	3 (20.0)	5 (41.7)	3 (27.3)	2 (25.0)	4 (57.1)	3 (50.0)	2 (33.3)	1 (20.0)	–	–	–
Cost	Total	**315**	**89**	**51**	**38**	**22**	**18**	**17**	**15**	**12**	**11**	**8**	**7**	**6**	**6**	**5**	**4**	**4**	**2**
	0–20 USD	**32 (10.1)**	5 (5.6)	8 (15.7)	4 (10.3)	–	4 (22.2)	2 (11.8)	1 (6.7)	1 (8.3)	2 (18.2)	–	–	2 (33.3)	1 (16.7)	–	1 (25.0)	–	1 (50.0)
	21–100 USD	**4 (1.3)**	–	–	–	2 (9.1)	1 (5.6)	–	–	–	–	1 (12.5)	–	–	–	–	–	–	–
	Over 100 USD	**14 (4.4)**	3 (3.4)	4 (7.8)	1 (2.6)	1 (4.5)	1 (5.6)	–	2 (13.3)	–	–	–	1 (14.3)	–	1 (16.7)	–	–	–	–
	Not specified/no costing yet	**265 (84.2)**	81 (91.0)	39 (76.5)	33 (87.2)	19 (86.4)	12 (66.7)	15 (88.2)	12 (80.0)	11 (91.7)	9 (81.8)	7 (87.5)	6 (85.7)	4 (66.7)	4 (66.7)	5	3 (75.0)	4	1 (50.0)
Study design	Total	**315**	**89**	**51**	**38**	**22**	**18**	**17**	**15**	**12**	**11**	**8**	**7**	**6**	**6**	**5**	**4**	**4**	**2**
	Randomized clinical trials	**12 (3.8)**	1 (1.1)	–	–	–	–	2 (11.8)	–	–	3 (27.3)	2 (25.0)	3 (42.9)	–	–	–	1 (25.0)	–	–
	Observational cohort studies/case–control studies	**102 (32.3)**	32 (36.0)	17 (33.3)	16 (41.0)	1 (4.5)	1 (5.6)	9 (52.9)	3 (20.0)	2 (16.7)	5 (45.5)	3 (37.5)	2 (28.6)	4 (66.7)	3 (50.0)	1 (20.0)	3 (75.0)	–	--
	Case series/case reports	**34 (10.8)**	7 (7.9)	9 (17.6)	7 (17.9)	3 (13.6)	2 (11.1)	1 (5.9)	2 (13.3)	–	–	–	–	–	1 (16.7)	2 (40.0)	–	–	–
	Diagnostic accuracy studies	**98 (31.0)**	34 (38.2)	22 (43.1)	10 (25.6)	4 (18.2)	7 (38.9)	1 (5.9)	6 (40.0)	4 (33.3)	1 (9.1)	2 (25.0)	1 (14.3)	1 (16.7)	2 (33.3)	1 (20.0)	–	2 (50.0)	–
	Qualitative studies	**5 (1.6)**	1 (1.1)	1 (2.0)	1 (2.6)	1 (4.5)	–	1 (5.9)	–	–	–	–	–	–	–	–	–	–	–
	Product/technical descriptions	**64 (20.6)**	14 (15.7)	2 (3.9)	4 (12.8)	13 (59.1)	8 (44.4)	3 (17.6)	4 (26.7)	6 (50.0)	2 (18.2)	1 (12.5)	1 (14.3)	1 (16.7)	–	1 (20.0)	–	2 (50.0)	2
Study population size	Total	**315**	**89**	**51**	**38**	**22**	**18**	**17**	**15**	**12**	**11**	**8**	**7**	**6**	**6**	**5**	**4**	**4**	**2**
	1–30	**84 (26.6)**	24 (27.0)	10 (19.6)	16 (41.0)	7 (31.8)	4 (22.2)	3 (17.6)	5 (33.3)	1 (8.3)	1 (9.1)	4 (50.0)	1 (14.3)	1 (16.7)	3 (50.0)	2 (40.0)	2 (50.0)	–	–
	31–100	–	–	–	–	–	–	–	–	–	–	–	–	–	–	–	–	–	–
	101–500	**124 (39.6)**	37 (41.6)	26 (51.0)	13 (35.9)	2 (9.1)	3 (16.7)	9 (52.9)	7 (46.7)	7 (58.3)	6 (54.5)	1 (12.5)	4 (57.1)	4 (66.7)	3 (50.0)	–	1 (25.0)	1 (25.0)	–
	501–1000	**4 (1.3)**	3 (3.4)	–	–	–	–	1 (5.9)	–	–	–	–	–	–	–	–	–	–	–
	>1000	**14 (4.4)**	5 (5.6)	4 (7.8)	1 (2.6)	–	–	1 (5.9)	–	2 (16.7)	–	–	–	–	–	–	–	1 (25.0)	–
	None/not specified	**89 (28.2)**	20 (22.5)	11 (21.6)	8 (20.5)	13 (59.1)	11 (61.1)	3 (17.6)	3 (20.0)	2 (16.7)	4 (36.4)	3 (37.5)	2 (28.6)	1 (16.7)	–	3 (60.0)	1 (25.0)	2 (50.0)	2
Study quality	Total	**315**	89	51	38	22	8	17	15	12	11	8	7	6	6	5	4	4	2
	−	**18**	4	9	2	1	0	0	1	0	0	0	0	0	0	1	0	0	0
	+	**64**	26	9	8	7	2	0	1	3	1	0	1	3	0	3	0	0	0
	++	**233**	59	33	28	14	16	17	13	9	10	8	6	3	6	1	4	4	2

Bold values are those for which the columns and rows correspond to totals, rather than to one specialty or characteristic.

^a^Not all totals add to 315 due to presence of more than one value in some papers.

### Epidemiology

Most studies described technologies to screen for, diagnose or monitor conditions within the clinical domains of cardiovascular medicine (89/315), ophthalmology and otorhinolaryngology (51/315), neurology (39/315), general medicine (22/315) and maternal and child health (17/315). Development of products occurred predominantly in North America (118/315), followed by Asia (77/315) and Europe (60/315).

Among those studies that were included in our review, the specific NCDs that were most frequently targeted by the study intervention, in order of descending frequency, were arrhythmias (*n* = 40), Parkinson’s disease (*n* = 20), retinal pathologies (*n* = 14), hearing loss (*n* = 13), melanoma and other skin cancers (*n* = 11), diabetes (*n* = 10), anemia (*n* = 7), and visual impairment (*n* = 7). Studies focusing on these conditions are summarized in Supplementary Table 3.

### Technology

The most popular device platform for studied technologies was the smartphone (253/315), followed by tablets (31/315) and “conventional” mobile phones (21/315), i.e., phones without internet capabilities. Applications were predominantly developed using Apple iOS (113/315) and Android (98/315), with 21 working across different operating systems. Very few were tailored specifically for BlackBerry (2/315), Windows (5/315), or other operating systems such as NetBeans, Tiny OS platform, or Symbian (3/315). In terms of connectivity, some of them required internet connection to work as intended (56/315), while most did not (258/315) and two did not specify. Similarly, a minority required a Bluetooth connection (59/315). However, the majority required the use of accessories (209/315), such as additional lenses to cameras and wired sensors to detect movement and electrical activity developed specifically for the technologies.

Due to the incipient nature of the technologies, costs were not readily available for the vast majority (266/315) of them. For products with price information (50/315), most cost less than 20 USD (32/50), followed by between 21 and 100 USD (4/50) and over 100 USD (14/50).

### Methodology

Most of the included publications focused on technologies in an advanced development stage, i.e., already validated and/or commercially available (211/315) (Supplementary Table 2), with the remaining studies describing prototypes (50/315), proof of concept/principle studies (20/315), validation tests (6/315), and pilots (6/315). 16 analyses did not specify the development stage of the products. Publications presenting applications that were already developed or available as described by the authors are summarized in Supplementary Table 4. There was wide variation in studies’ assessment of technology depending on the developmental stage: early-development innovations were assessed through product or technical descriptions (65/315), whereas those further in development were subjected to observational cohort or case–control studies (102/315) or diagnostic accuracy studies (98/315), and a few of them tended to be evaluated via more rigorous experimental methodologies (12/315), such as randomized clinical trials. A small number evaluated qualitative aspects of the product, assessing the attitudes of healthcare workers towards mHealth applications (5/315). To assess the studied innovations, most analyses recruited cohorts between 101 and 500 subjects (125/315), followed by several over 1000 participants (14/315) and those with fewer than 30 subjects (84/315). Some publications did not need to specify a sample size due to the study design used (89/315).

### NCDs of high importance

Table 2 summarizes the 24 studies that focused on one of the ten diseases responsible for the most disability-adjusted life years (DALYs) lost in LMICs in 2019:^24^ ischemic heart disease, chronic obstructive pulmonary disease, neonatal preterm birth, Type 2 diabetes, lower back pain, ischemic stroke, age-related hearing loss, falls, and other musculoskeletal concerns. The uses of such mHealth applications ranged from diagnosing and screening to monitoring these ten disorders. Nearly all of these interventions were smartphone-based (20/24) and most were already available or fully developed (18/24). On the whole, authors of the studies did not specify the cost of the technologies; for those that did, most were under 5 USD (5/6). The majority of mHealth products reviewed were developed either exclusively in high-income countries (HICs, as defined by the World Bank 2021 country classifications) (13/24), or HICs in association with LMIC organizations (3/24). Eight were developed exclusively in LMICs (8/24).

**Table 2:** Summary of studies focusing on one of the top ten global DALY-contributing diseases (*N* = 24).

Disease	Title	Authors	Disease RF	Clinical domain	Aim	Type of intervention	Mobile device	OS	Study population	Methods	Stage of development	Cost	Year	Author affiliation
Ischemic Heart Diseases	Feasibility of combining serial smartphone single-lead electrocardiograms for the diagnosis of ST-elevation myocardial infarction: Smartphone ECG for STEMI Diagnosis	Muhlestein et al.	ST-elevation myocardial infarction (STEMI)	Cardiology	Diagnosis	Smartphone-based ECG	Smartphone	Not specified	Subjects were enrolled from 5 international sites.	Experimental	Developed	Not specified	2020	USA
	Smartphone ECG for evaluation of STEMI: results of the ST LEUIS Pilot Study	Muhlestein et al.	STEMI	Cardiology	Monitoring	Smartphone application	iPod	iOS	Patients for whom the hospital STEMI protocol was activated	Observational Cohort Studies/case–control studies	Prototype	Not specified	2015	Multinational (USA, Argentina)
	Plasmonic ELISA for Sensitive Detection of Disease Biomarkers with a Smart Phone-Based Reader	Quanli Yang	Acute myocardial infarction	Cardiology	Screening	Smartphone application	Smartphone	Android and iOS	Serum samples were collected from the Guangzhou Overseas Chinese Hospita	Technical description	Developed	About two dollars	2018	China
Chronic Obstructive Pulmonary Disease	The utility of hand-held mobile spirometer technology in a resource-constrained setting.	Du Plessis et al.	Chronic respiratory diseases	Pulmonary Medicine	Screening	Smartphone application	Smartphone	Not specified	Consecutive patients and healthy volunteers	Observational Cohort Studies/case–control studies	Developed	Not specified	2019	South Africa
Neonatal Preterm Birth	Mobile phones for retinopathy of prematurity screening in Lagos, Nigeria, sub-Saharan Africa	Tunji S. Oluleye et al.	Retinopathy of Prematurity	Ophthalmology	Screening	Smartphone application	Smartphone	iOS	Preterm infants with birthweight of less than 1.5 kg or gestational age of less than 32 weeks	Technical testing	Available	Not specified	2016	Nigeria
	MII RetCam assisted smartphone-based fundus imaging for retinopathy of prematurity	Lekha et al.	Retinopathy of prematurity	Ophthalmology	Diagnosis/ Monitoring	Smartphone add on	Smartphone	iOS	All the preterm babies subjected to smartphone-based fundus imaging as part of ROP screening from September 2017 to November 2018	Retrospective observational	Developed	MII RetCam device costs USD 380/–	2019	India
Diabetes Type 2	Mobile communication using a mobile phone with a glucometer for glucose control in Type 2 patients with diabetes: as effective as an Internet-based glucose monitoring system	Cho et al.	Diabetes type 2	Endocrinology	Monitoring	Smartphone add on	Mobile phone	Not specified	Type 2 diabetes patients	Experimental	Developed	Not specified	2009	Republic of Korea
	Reusable electrochemical glucose sensors integrated into a smartphone platform.	Bandodkar et al.	Diabetes	Endocrinology	Monitoring	Smartphone-based reusable glucose meter	Smertphone	Android	NA	Technical testing	Prototype	Not specified	2018	USA
	Evaluation of a mobile-phone telemonitoring system for glycaemic control in patients with diabetes	Istepanian et al.	Diabetes	Endocrinology	Monitoring	Mobile phone-based system	Motorola A-100 mobile phone	Android	Patients with complicated diabetes	Experimental	Not specified	Not specified	2009	United Kingdom
	Ultrabright Polymer-Dot Transducer Enabled Wireless Glucose Monitoring via a Smartphone	Sun et al.	Diabetes	Endocrinology	Monitoring	Smartphone application	Smartphone-Huawei Mate 9	Android	Balb/c nude mice (Vital River Laboratories, Beijing, China). 8-week-old female mice	Experimental	In vitro and in vivo studies	Not specified	2018	China
	Real time monitoring of glucose in whole blood by smartphone	Erenas et al.	Diabetes	Endocrinology	Monitoring	Combined thread-paper microfluidic device	Sony DSC-HX300 digital camera, a Samsung Galaxy S5 smartphone, a Samsung Galaxy Tab A tablet, and a Motorola Moto G4 Play smartphone	Android	None	Technical testing	Developed	Not specified	2019	Multinational (Spain, USA)
	Smartphone-based noninvasive salivary glucose biosensor	Soni and Jha	Diabetes	Endocrinology	Diagnosis/Screening	Smartphone application	Smartphone	Android	Subjects between age group 20–80 years at Outpatient Department of Indian Institute of Technology Delhi hospital, New Delhi	Experimental	Developed	Not specified	2017	India
	Noninvasive blood glucose monitor based on spectroscopy using a smartphone.	Dantu et al.	Diabetes	Endocrinology	Monitoring	Noninvasive blood glucose monitor	Smartphone	Android	Human subjects who drank Cola beverage of 50 g sugar	Observational Cohort Studies/case–control studies	Developed	Not specified	2014	USA
Low Back Pain	mDurance: A Novel Mobile Health System to Support Trunk Endurance Assessment.	Banos et al.	Low back pain	Sports medicine	Monitoring	Wearable and mobile devices	Smartphone	Android	Case study	Technical testing	Developed	Not specified	2015	Multinational (Republic of Korea, Spain)
Ischemic Stroke	Smartphone electrographic monitoring for atrial fibrillation in acute ischemic stroke and transient ischemic attack	Tu et al.	Paroxysmal atrial fibrillation	Cardiology	Monitoring	Smartphone application	Smartphone	Android and iOS	Patients with ischemic stroke or transient ischemic attack (TIA) without known AF, Age > 18 years	Prospective cohorts	Proof of principle	Not specified	2017	Multinational (Australia, China)
Other musculoskeletal	Reliability Analysis of a Smartphone-aided Measurement Method for the Cobb Angle of Scoliosis	Qiao et al.	Adolescent Idiopathic Scoliosis	Traumatology	Diagnosis	Smartphone application	Smartphone	iOS	Posteroanterior radiographs of adolescent idiopathic scoliosis patients with thoracic scoliosis	Observational Cohort Studies/case–control studies	Developed	Not specified	2011	China
	Screening of scoliosis in school children in Tehran: The prevalence rate of idiopathic scoliosis	Shahrbanoo Kazem et al.	Scoliosis	Orthopedics	Screening	Smartphone application	Smartphone	iOS	School children in Tehran	Experimental	Available	$4.99	2018	Iran
	Evaluation of an apparatus to be combined with a smartphone for the early detection of spinal deformities.	Driscoll et al.	Spinal deformities	Orthopedics	Diagnosis	Smartphone application	Smartphone	iOS	Adolescents with adolescent idiopathic scoliosis	Observational Cohort Studies/case–control studies	Developed	Not specified	2014	Canada
	Validation of a scoliometer smartphone app to assess scoliosis.	Franko et al.	Scoliosis	Orthopedics	Diagnosis	Smartphone application	Smartphone	iOS	Measurements	Experimental	Developed	The cost of the application ($0.99) and manufacturing the custom part were <$25, and when purchased in bulk would cost <$5/unit.	2012	USA
Age-related hearing loss	Extended High-Frequency Smartphone Audiometry: Validity and Reliability.	Bornman et al.	Age-related hearing loss, noise-induced hearing loss (NIHL) and ototoxicity	Otorhinolaryngology	Screening	Smartphone application	Smartphone	Android	“Participants were recruited from adults attending the Audiology Department at Dr. George Mukhari Hospital, GaRankuwa, South Africa and from the University of Pretoria”	Observational Cohort Studies/case–control studies	Developed	Not specified	2019	Multinational (Australia, South Africa)
	Implementation of uHear™—an iOS-based application to screen for hearing loss—in older patients with cancer undergoing a comprehensive geriatric assessment	Michelle et al.	Presbycusis	Otorhinolaryngology	Screening	Smartphone application	iPod, iPhone, iPad	iOS	Older patients with cancer at the radiotherapy and oncology departments of the General Hospital Groeninge (Kortrijk, Belgium) from December 2014 till June 2015	Observational Cohort Studies/case–control studies	Available	Not specified	2016	Belgium
	Application-Based Hearing Screening in the Elderly Population	Leonid et al.	Presbycusis (Hearing loss)	Otorhinolaryngology	Screening	Smartphone application	Tablet	iOS	Patients 65 years of age or older hospitalized for any reason in an internal medicine department	Experimental	Available	Free	2017	USA
	Smartphone-based audiometric test for screening hearing loss in the elderly.	Abu-Ghanem et al.	Hearing loss	Otorhinolaryngology	Screening	Smartphone application	Smartphone—iPhone and Tablet—iPod, iPad	iOS	Subjects aged 84.4 ± 6.73 years (mean ± SD) were recruited.	Observational Cohort Studies/case–control studies	Available	Free	2015	Israel
Falls	iFall: An android application for fall monitoring and response	Sposaro et al.	Fall	Geriatrics	Monitoring	Smartphone application	Smartphone	Android	None	Technical description	Prototype	Not specified	2009	USA

## Discussion

Our systematic review of mHealth interventions for NCDs relevant to LMIC healthcare providers identified several important characteristics of this current landscape. We found that most of these interventions were relatively affordable (in the small number of cases where cost data were provided), generally at an advanced stage of development, and designed to be employed using smartphone devices while favoring Apple iOS as the preferred operating system, consistent with the fact that most of the interventions were developed in HICs—albeit occasionally in partnerships with LMIC organizations. Concerningly, most applications (92%) focused on diseases other than the ten responsible for the most DALYs lost globally, and only a small minority employed rigorous randomized clinical trial methodology.

In further detail, our first key finding was that most of the reported technologies were developed in North America, Europe, and Asia—specifically from HICs within these continents. Seldom were LMIC institutions listed as leading the research of the mobile applications identified in our search; even when they were, it was typically in partnership with HIC organizations. This phenomenon has previously been detailed by analyses^25,26^ which conclude that the poor local health research capacities of LMIC institutions are due to uneven power relations between them and international funders, weak links between research policy and practice, and the lack of a systems approach to research capacity development. In fact, this observation has been called the “10/90 gap”, whereby LMICs possess ninety percent of the global disease burden but only ten percent of global funding for health. This disparity is critical, as many mHealth applications created in high-income country settings may not be applicable for use in LMICs due to factors such as local disease prevalence and availability of diagnostics and therapeutics in low-resource settings. “North-South Partnerships”, project-specific collaborations between HIC and LIC research institutions which were identified in our study, may hold promise for promoting more sustainable research models, though they remain controversial^25^.

The disconnect between the HICs generating mHealth and LMICs utilizing mHealth becomes more apparent when examining applications’ choice of operating systems. Even though the Android operating system is the most popular in the world, holding ~73–87% of market share^27,28^, our study found that Apple iOS-based mobile technologies are almost twice as prevalent as Android-based technologies. This incongruence in operating systems may represent another access barrier: people can only use tools that are supported by their devices.

Most concerningly, we found that only 24 of the 315 technologies included in our sample focus on the ten diseases responsible for the greatest number of DALYs lost globally. Many of these conditions have historically relied on expensive specialized equipment for screening, diagnosis, and monitoring; for example, many cardiovascular diseases require such tools as electrocardiograms, echocardiograms, and rhythm monitors for optimal management^29^, yet these technologies can cost thousands to tens of thousands of dollars. This represents a cost-prohibitive barrier to access for many LMICs, particularly in the setting of compounding systemic issues such as sociopolitical instability, low health expenditures, and corruption^30–32^. As mHealth technologies hold promise for lower-cost management of these illnesses of great public health importance, the relatively low number of technologies devoted to innovatively disrupting these fields remains disappointing. Rather, the focus on disorders that affect quality of life in HICs appears to be reflected in the choice of target diseases selected by their investigators. It is also noteworthy that researchers seldom attempted to extend clinician-oriented findings to questions of specific interest to policymakers or program managers. We believe an effort to make these findings more policy-relevant is one of several potential steps forward (Box 1).

Our results further reveal that smartphones are the main device for mHealth development and highlight the importance of connectivity (both device-to-device and to the internet) in the functionality of mobile technologies. This has important implications for ongoing efforts to equip CHWs with tools to maximize their essential role in resource-constrained settings—i.e., smartphones, rather than tablets, may be more useful devices.

Lastly, our results suggest that the overall validation process of these mHealth technologies is still in its early stages. Whereas many of the technologies are already available commercially, most of the testing and evaluation has been done through pretesting and pilot studies, without the rigor of randomized controlled trials to determine their clinical efficacy compared to current standards of care. That said, a significant number of these innovations have been subjected to initial diagnostic accuracy studies with modest sample sizes, often in comparison to clinical gold standards, paving the way for future analyses of their effect on measurable patient outcomes.

Box 1. Way forward from the present study

- Generation of a more robust body of evidence regarding the accuracy, feasibility, and cost-effectiveness of mHealth technologies addressing diseases with the highest public health burdens to inform potential policymaking efforts
- Greater involvement of LMIC stakeholders from the earliest stages of mHealth technology research and development to ensure usability and deployment of technologies for end users in resource-constrained settings
- Efforts toward transferring technology and knowledge between HICs and LMICs and between diseases to maximize the impact of a given technology

### Limitations

Our present study has several limitations. First, the considerable design and population heterogeneity of the publications analyzed precluded the systematic assessment of study quality (by validated tools such as the GRADE framework^33^). We thus attempted to quantify the quality of evidence in simpler terms by extracting cohort size, general study design, and measurement instruments. Future analyses focusing on more granular subgroups within our study can clarify these findings. Similarly, a fine-grained technical analysis of the phase of product development was beyond the scope of the present work. Hence, our lack of granularity in the appraisal of early-development technologies, e.g., differentiating between diffusion and refinement phases, is also a limitation and area for potential further investigation. Similarly, we have categorized development stage as “advanced” for technologies that were validated, commercially available, or both, a simplification given that not all commercially available technologies have been validated. Finally, the studies included in this review were not necessarily targeted or designed for the populations in LMICs, and hence these findings represent a bare minimum for provider-focused mHealth technologies potentially usable for these conditions. Demonstrating that such technology transfer is feasible will be essential to realize the promise of these technologies.

Our results indicate that mHealth holds promise to equip LMIC healthcare providers with powerful tools to improve population NCD health. However, widespread implementation of these technologies still faces barriers, in particular unbalanced health research development between HICs and LMICs that translates into a disconnect between developer and user software choice, priority diseases being addressed, missing product cost reporting, and lack of rigorous randomized clinical trials to detect improvements in patient outcome. These limitations must be acknowledged and addressed for the full potential of mHealth to be achieved in LMICs.

## Methods

### Search strategy

We searched for English-language studies published between January 2007 and October 2019 in the following indexing databases: Cochrane Central (searched on September 30th, 2019), PubMed (searched on October 7th, 2019), and Web of Science (searched on October 7th, 2019). Keywords and medical subject headings (MeSH) used included “smartphones”, “tablets”, “diagnosis”, “screening”, and “monitoring”. The full list of search terms for each database are shown in Supplementary Table 1.

No restrictions were placed on study design, sample size, or publication type. Additionally, the reference lists of all included studies and relevant review articles and commentaries were screened for additional references. The search process is graphically summarized in Fig. 1. Our systematic review was registered in The International Prospective Register of Systematic Reviews (PROSPERO; Registration number: CRD42020193945)^34^.

### Inclusion and exclusion criteria

Titles and abstracts were screened for relevance, and then the full‐text versions of retrieved publications were assessed using the following three inclusion criteria: (i) the technology reported must be mobile phone- or tablet-based, (ii) the technology reported must be able to screen for, diagnose, or monitor a disease, and (iii) the disease the technology is designed to address must be an NCD of public health importance for LMICs, defined as conditions that are estimated to cause more than 1% of deaths in any 5-year-age group in the general population or among neonates, or diseases that have a prevalence of greater than 0.1% in any 5-year-age group in the general population or among neonates. The Global Burden of Disease Project’s 2019 estimates were used for the assessment as to whether a condition is an NCD of public health importance in LMICs^35^.

### Data extraction

The following data were extracted from each included article: author(s), title, disease or risk factor, clinical domain by MeSH^36^, intervention name, intervention type, purpose and aim of the intervention, target population, type of mobile device utilized, type of software, operating system used by intervention, study population, sample size, study methods, stage of development, cost, country of development and/or testing (based on the authors’ institutional affiliations and/or the study population country of residence), year of publication, and a summary of the tool. These data were extracted qualitatively using Microsoft Excel (Redmond, WA).

### Analysis

The extracted data were synthesized into three themes based on study characteristics: epidemiology, technology, and methodology. Supplementary Table 2 describes these themes and associated categories, subcategories, and definitions. We subsequently constructed tables crossing clinical domains with all the subthemes to identify trends across the studies.

Due to the large degree of heterogeneity in study designs, outcome measures, and reporting of outcomes, meta-analysis techniques were unable to be used to further summarize the studies.

### Reporting summary

Further information on experimental design is available in the Nature Research Reporting Summary linked to this paper.

## Supplementary information

Supplemental Tables A1-A4

Reporting Summary Checklist

## Data Availability

The authors confirm that the data supporting the findings of this study are available within the article and its Supplementary Material. Any further data analysis information is available from the corresponding author by request.
