# Supplementary material for: A systematic review of healthcare provider-targeted mobile applications for non-communicable diseases in low- and middle-income countries
Source: NPJ Digit Med. 2022 Jul 19;5:99. doi: 10.1038/s41746-022-00644-3 (PMC9296618; doi:10.1038/s41746-022-00644-3)
Supplement: Supplementary file 1 — Supplemental Tables A1-A4 [file 41746_2022_644_MOESM1_ESM.pdf]

Supplementary Information for “A systematic review of healthcare provider-targeted mobile applications for non-communicable diseases in low- and middle-income countries”

Supplementary Table 1. Search Strategies

Date of search	Database	Dates range	Search strategy
**07.10.2019**	Pubmed	2007-2019	2007:2019 [dp] (smartphone [mesh] OR smartphone [tiab] OR smart phone* [tiab] OR mobile phone* [tiab] OR mobile device* [tiab] OR cell phone* [tiab] OR tablet [mesh] OR tablet [tiab] OR tablets [tiab] OR "portable device" [tiab] OR ipad OR iphone* OR "android" OR iPhone Operating System) AND ("app" [tiab] OR "apps" [tiab] OR application [tiab] OR applications [tiab] OR mobile application* [tiab] OR software [tiab] OR tool [tiab]) AND (diagnosis [mesh] OR diagnose [tiab] OR diagnosed [tiab] OR diagnosis [tiab] OR diagnoses [tiab] OR diagnostic [tiab] OR diagnostics [tiab] OR screening [mesh] OR screening [tiab] OR screen [tiab] OR screened [tiab] OR screens [tiab] OR monitor [mesh] OR monitor [tiab] OR monitors [tiab] OR monitoring [tiab] OR monitored [tiab])
**07.10.2019**	Web of Science	2007-2019	(smartphone [mesh] OR smartphone OR smart phone* OR mobile phone* OR mobile device* OR cell phone* OR tablet [mesh] OR tablet OR tablets OR ipad OR iphone* OR "android" OR iPhone Operating System) AND TOPIC: ("app" OR "apps" OR application OR applications OR mobile application* OR software OR tool) AND TOPIC: (diagnosis [mesh] OR diagnose OR diagnosed OR diagnosis OR diagnoses OR diagnostic OR diagnostics OR screening [mesh] OR screening OR screen OR screened OR screens OR monitor [mesh] OR monitor OR monitors OR monitoring OR monitored)<br>Timespan: 2007-2019.
**30.09.2019**	Cochrane Central	2007-2019	((smartphone OR smart phone* OR mobile phone* OR mobile device* OR cell phone* OR tablet OR tablets OR iphone or iphones OR "android" OR iPhone Operating System OR ipad)):ti,ab,kw AND (("app" OR "apps" OR application OR applications OR mobile application* OR software OR tool)):ti,ab,kw AND ((diagnose OR diagnosed OR diagnosis OR diagnoses OR diagnostic OR diagnostics OR screening OR screen OR screened OR screens OR monitor OR monitors OR monitoring OR monitored)):ti,ab,kw

Supplementary Table 2. Themes and Categories

THEMES	SUBTHEMES	CATEGORIES	DEFINITIONS OR SUBCATEGORIES
**EPIDEMIOLOGY**	Medical Specialties	Cardiology	Medical specialty concerned with the study of the heart, its physiology, and its functions.
		Ophthalmology and Otorhinolaryngology	A surgical specialty concerned with the structure and function of the eye and the medical and surgical treatment of its defects and diseases. / A surgical specialty concerned with the study and treatment of disorders of the ear, nose, and throat
		Neurology	A medical specialty concerned with the study of the structures, functions, and diseases of the nervous system.
		General Medicine	
		Maternal and Child Healthcare	The medical specialties concerned with the physiology and disorders primarily of the female genital tract, as well as female endocrinology and reproductive physiology, and maintaining health and providing medical care to children from birth to adolescence.
		Dermatology	A medical specialty concerned with the skin, its structure, functions, diseases, and treatment.
		Endocrinology	A subspecialty of internal medicine concerned with the metabolism, physiology, and disorders of the endocrine system.
		Hematology	A subspecialty of internal medicine concerned with morphology, physiology, and pathology of the blood and blood-forming tissue// the dynamics of blood flow.
		Oncology	A subspecialty of internal medicine concerned with the study of neoplasms.
		Psychiatry	The medical science that deals with the origin, diagnosis, prevention, and treatment of mental disorders.
		Orthopedics and Traumatology	A specialty which utilizes medical, surgical, and physical methods to treat and correct deformities, diseases, and injuries to the skeletal system, its articulations, and associated structures.
		Pulmonary Medicine	A subspecialty of internal medicine concerned with the study of the respiratory system. It is especially concerned with diagnosis and treatment of diseases and defects of the lungs and bronchial tree.
		Nutrition and Sports Medicine	The field of medicine concerned with physical fitness and the diagnosis and treatment of injuries sustained in exercise and sports activities, as well as nutrition processes during exercise and athletic performance, nutritional requirements of athletes and the relationship between nutritional status and nutrition disorders in athletes.
		Surgery and Anesthesiology	Involves various branches of surgical practice limited to specialized areas in the human anatomy, alongside the study of anesthetics and anesthesia.
		Rheumatology	A subspecialty of internal medicine concerned with the study of inflammatory or degenerative processes and metabolic derangement of connective tissue structures which pertain to a variety of musculoskeletal disorders, such as arthritis.
		Allergology and Immunology	A medical specialty concerned with the hypersensitivity of the individual to foreign substances and protection from the resultant infection or disorder.
		Nephrology and Urology	Subspecialities concerned with the anatomy, physiology, and pathology of the kidney and concerned with the study, diagnosis, and treatment of diseases of the urinary tract in both sexes, and the genital tract in the male.
**TECHNOLOGY**	Type of Mobile Device*	Armband/Smartwatch	Wearable computers in the forms of a watch or a band usually wrist worn.
		Smartphones	A cell phone that includes additional software functions (such as email or an Internet browser)
		Mobile Phones	A portable, usually cordless, telephone for use in a cellular system
		Tablets	A mobile computing device that has a flat, rectangular form like that of a magazine or pad of paper, that is usually controlled by means of a touch screen, and that is typically used for accessing the Internet, watching videos, playing games, reading electronic books, etc.
		iPod Devices	A small electronic device for playing and storing digital audio and video files, proprietary of Apple Inc.

		PC	A general-purpose computer equipped with a microprocessor and designed to run especially commercial software (such as a word processor or Internet browser) for an individual user
		Other wireless devices	Blue Box device, Bluetooth enabled monitoring system, digital camera, UWB P410 Radar Module, wireless bio patch
	Development Stage	Proof of Concept/Proof of Principle	Evidence that shows that a (…) design idea, technology etc. will work, usually based on an experiment or a pilot project.
		In development	Technology in the process of being prepared, developed or completed.
		Prototype	A first full-scale and usually functional form of a new type or design of a construction
		Pilot	A small-scale preliminary study conducted to evaluate feasibility, duration, cost, adverse events, and improve upon the study design prior to performance of a full-scale research project or technology deployment.
		Validation Trial/Test in Clinical Trial	A scientifically controlled study of the safety and effectiveness of a therapeutic agent (such as a drug or vaccine) using consenting human subjects
		Available/Developed	Technologies that have undergone development and trials and are currently available to be used by practitioners, including those available at virtual application stores for purchase or free download.
		Not specified	Study does not state the stage of development.
	Operating System	iPhone Operating System	Mobile operating system created and developed by Apple Inc. exclusively for its hardware.
		Android	Mobile operating system based on a modified version of the Linux kernel and other open-source software, designed primarily for touchscreen mobile devices such as smartphones and tablets
		Windows	A group of several proprietary graphical operating system families, all of which are developed and marketed by Microsoft and targeted to different devices, ranging from Personal Computers to Mobile Phones.
		Blackberry	A proprietary mobile operating system developed by Canadian company BlackBerry Limited for its BlackBerry line of smartphone handheld devices.
		MultiPhone Operating System	Applicable to technologies that work on multiple operating systems
		Others	Includes HTML5, MIDiet Implementation of JM2E using NetBeans 5.5, Symbian, Tiny OS Platform
		Not specified	Not detailed in the study
	Use of Accessories	Yes/No	Use or not of accessories additional to the mobile technology to achieve its purpose (i.e., electrodes, cellphone attachments, small screens, blood pressure cuffs, additional light sources, straps or bands, lenses, headphones)
	Cost	Ranges of $	0-20 USD, 21-100 USD, >100 USD, or Not specified or not costing assigned yet. Some costs in different currencies were converted to USD dollars using Google at April 2021 rates of conversion.
**METHODOLOGY**	Author Affiliation	North America	Canada, USA
		South America	Brazil, Colombia
		Europe	Austria, Belgium, Denmark, England, Finland, France, Germany, Greece, Hungary, Ireland, Italy, Lithuania, Netherlands, Poland, Portugal, Spain, Sweden, Switzerland, Turkey, United Kingdom
		Africa	Cameroon, Madagascar, Nigeria, Rwanda, South Africa, Uganda, Zambia
		Asia	Bangladesh, Bhutan, China, Hong Kong, India, Indonesia, Iran, Israel, Japan, Nepal, Republic of Korea, Singapore, South Korea, Taiwan, United Arab Emirates
		Oceania	Australia, New Zealand
		Multinational	Including several countries in the same study, either as collaborators or as study settings.
	Year of Publication	Year Ranges	2006-2008, 2009-2011, 2012- 2014, 2015-2017, 2018- 2020

	Study Design	Randomized Clinical Trials	A study design that randomly assigns participants into an experimental group or a control group. As the study is conducted, the only expected difference between the control and experimental groups in a randomized controlled trial (RCT) is the outcome variable being studied.
		Observational Cohort Studies/Case Control Studies	Studies where researchers observe the effect of the studied technology without trying to change who is or isn't exposed to it.
		Case Series/Case Reports	A detailed report of the implementation of a technology in the diagnosis, monitoring or screening of an individual patient. A case series is group of case reports involving patients that were assisted in the diagnostic process by the same technology.
		Diagnostic Accuracy Study	A study that provides evidence on how well a technology correctly identifies or rules out disease and informs subsequent decisions about treatment for clinicians, their patients, and healthcare providers.
		Qualitative	Studies that collected and analyzed non-numerical data (e.g., text, video, or audio) to understand concepts, opinions, or experiences.
		Technical Description/Testing	A technical description describes a technology in terms of its function, organization, parts and details, without using a study design framework to do so.
**Sources: NCBI Mesh Medical Specialties[1], "Research Design: Qualitative, Quantitative, and Mixed Methods Approaches" Book[2].**			

'An algorithm for the classification of study designs to assess diagnostic, prognostic, and predictive test accuracy in systematic reviews'[3]

1. Medicine - MeSH - NCBI [Internet]. [cited 2021 May 13]. Available from: https://www.ncbi.nlm.nih.gov/mesh/?term=medical+specialties

2. Creswell JW. Research Design: Qualitative, Quantitative, and Mixed Methods Approaches. SAGE Publications; 2009. 297 p.

3. Mathes T, Pieper D. An algorithm for the classification of study designs to assess diagnostic, prognostic, and predictive test accuracy in systematic reviews. Systematic Reviews. 2019 Sep 3;8(1):226.

Supplementary Table 3. Studies that include 10 most common diseases in database (n=121)

Diseases	Title	Authors	Disease RF	Clinical Domain	Aim	Type of Intervention	Mobile	Operating System	Study Population	Methods	Stage of Development	Cost	Year	Author Affiliation
Arrhythmias (N=40)	Pit-a-Pat: A Smart Electrocardiogram System for Detecting Arrhythmia.	Juyoung Park, Kuyeon Lee, Kyungtae Kang	Arrythmia	Cardiology	Monitoring	Mobile application	Smartphone	Android	MIT-BIH Arrythmia database	Technical Testing	Developed	Not specified	2015	South Korea
	Diagnostic Performance of a Smart Device With Photoplethysmography Technology for Atrial Fibrillation Detection: Pilot Study (Pre-mAFA II Registry).	Fan, Y. et al.	Atrial Fibrillation	Cardiology	Diagnosis	Smartphone application	Smartphones (HUAWEI Mate 9, HUAWEI Honor 7X), smart bands (HUAWEI Band 2)	Android	Consecutive inpatients were recruited from the Chinese People's Liberation Army General Hospital (Beijing, China) from March 15 to April 1, 2018	Pretest/Posttest	Developed	Not specified	2019	China
	Atrial Fibrillation Detection via Accelerometer and Gyroscope of a Smartphone	Lahdenoja, O. et al.	Atrial Fibrillation	Cardiology	Diagnosis	Smartphone application	Sony X-peria Z-series, Samsung Galaxy S5	Android	AFib patients and healthy controls	Experimental	Developed	Not specified	2016	Finland
	Real-time ECG monitoring and arrhythmia detection using Android-based mobile devices.	Gradl, S. et al.	Arrythmia	Cardiology	Monitoring	Mobile application	Smartphone - Samsung GT-I9000, Samsung GT-N7000, HTC Wildfire S A510e	Android	MIT-BIH Arrhythmia and the MIT-BIH Supraventricular databases.	Experimental	Prototype	Not specified	2014	Germany
	Smartphone electrographic monitoring for atrial fibrillation in acute ischemic stroke and transient ischemic attack	Tu, H. et al	Paroxysmal atrial fibrillation	Cardiology	Monitoring	Smartphone application	Smartphone	Android and iPhone Operating System	Patients with ischemic stroke or transient ischemic attack (TIA) without known AF, Age>18 years	Experimental	Proof of principle	Not specified	2017	Multinational
	Contact-Free Screening of Atrial Fibrillation by a Smartphone Using Facial Pulsatile Photoplethysmographic Signals	Bryan P. Yan, et al	Atrial fibrillation	Cardiology	Screening	Smartphone app	Smartphone	iPhone Operating System	Sample of 217 subjects	Experimental	Developed	Not specified	2018	United States
	Diagnostic Accuracy of a Novel Mobile Phone Application for theDetection and Monitoring of Atrial Fibrillation	Guy Rozen	Atrial fibrillation	Cardiology	Screening	Smartphone application	Smartphone	iPhone Operating System	Patients with atrial fibrillation	Experimental	Developed	Not specified	2018	United States
	Atrial fibrillation screening in pharmacies using an iPhone ECG: a qualitative review of implementation	Nicole Lowres, Ines Krass, Lis Neubeck et al	Atrial fibrillation	Cardiology	Screening	Smartphone application	Smartphone	iPhone Operating System	Pharmacists that had been present during a previous study called SEARCH AF, from 10 pharmacies in Sydney, Australia.	Experimental	Available	Not specified	2015	Australia
	Passive Detection of Atrial Fibrillation Using a Commercially Available Smartwatch	Tison, G.H. et al.	Atrial fibrillation	Cardiology	Diagnosis	Mobile application	Apple watches	iPhone Operating System	Health eHeart Study participants with an Apple Watch	Experimental	Developed	Not specified	2018	United States
	Self-monitoring for atrial fibrillation recurrence in the discharge period post-cardiac surgery using an iPhone electrocardiogram	Lowres, N. et al.	Atrial fibrillation	Cardiology	Monitoring	Smartphone-based wireless single-lead ECG (SL-ECG)	Smartphone	iPhone Operating System	Patients with POAF following cardiac surgery	Observational	Developed	Not specified	2016	Australia

iPhone ECG application for community screening to detect silent atrial fibrillation: a novel technology to prevent stroke	Lau, J. et al.	Atrial fibrillation	Cardiology	Screening	Smartphone application	Smartphone	iPhone Operating System	Learning set and validation set of patients	Pretest/Posttest	Developed	Not specified	2013	Australia
Feasibility and cost-effectiveness of stroke prevention through community screening for atrial fibrillation using iPhone ECG in pharmacies. The SEARCH-AF study	Lowres, N. et al.	Atrial fibrillation	Cardiology	Screening	Smartphone application	Smartphone	iPhone Operating System	Pharmacy customers aged ≥65 years	Observational	Developed	Not specified	2014	Multinational
Screening for atrial fibrillation in 13 122 Hong Kong citizens with smartphone electrocardiogram	Chan, N. & Choy, C.	Atrial fibrillation	Cardiology	Screening	Smartphone-based wireless single-lead ECG (SL-ECG)	Smartphone	iPhone Operating System	Hong Kong citizens	Experimental	Developed	Not specified	2016	China
Diagnostic Accuracy of a Novel Mobile Phone Application for the Detection and Monitoring of Atrial Fibrillation	Rozen, G. et al.	Atrial fibrillation	Cardiology	Diagnosis/ Monitoring	Smartphone application	Smartphone	iPhone Operating System	Patients	Experimental	Developed	Not specifief	2018	Multinational
Can smartphone wireless ECGs be used to accurately assess ECG intervals in pediatrics? A comparison of mobile health monitoring to standard 12-lead ECG.	Gropler, M. et al.	Arrythmia	Cardiology	Monitoring	Wireless mobile health (mHealth) device	Smartphone	iPhone Operating System	Pediatric outpatients age <18 years presenting for cardiology clinic visits. 3 groups based on age: 0–5 years, 6–10 years, and 11–18 years.	Experimental	Developed	Not specified	2018	United States
A Smartphone Application to Diagnose the Mechanism of Pediatric Supraventricular Tachycardia.	Ferdman, D., Liberman, L., Silver, E.	Pediatric Supraventricular tachycardia	Cardiology	Diagnosis	Smartphone application	Smartphone	iPhone Operating System	Pediatric patients undergoing an ablation for SVT	Experimental	Developed	Not specified	2015	United States
A single center randomized, controlled trial investigating the efficacy of a mHealth ECG technology intervention to improve the detection of atrial fibrillation: the iHEART study protocol	Hickey, K.T. et al.	Atrial fibrillation	Cardiology	Diagnosis	Smartphone application	Smartphone	iPhone Operating System	Participants with a recent history of atrial fibrillation	Experimental	Developed	Not specified	2016	United States
Smart detection of atrial fibrillation	Krivoshei, L. et al.	Atrial fibrillation	Cardiology	Screening	Smartphone application	Smartphone	iPhone Operating System	Consecutive in- and outpatients at the University Hospital Basel	Experimental	Developed	Not specified	2016	Mulinational
Detection of atrial fibrillation with a smartphone camera: first prospective, international, two-centre, clinical validation study (DETECT AF PRO)	Brasier, N. et al.	Atrial fibrillation	Cardiology	Diagnosis	Smartphone camera-based application	Smartphone	iPhone Operating System	In-house patients with presumed AF and matched controls SR	Experimental	Developed	Not specified	2018	Multinational
Mobile Phone–Based Use of the Photoplethysmography Technique to Detect Atrial Fibrillation in Primary Care: Diagnostic Accuracy Study of the FibriCheck App	Proesmans, T. et al.	Atrial fibrillation	Cardiology	Screening	Mobile phone application	Smartphone	iPhone Operating System	Patients aged 65 years and above, with or without a known history of AF, were recruited from 17 primary care facilities. Patients with an active pacemaker	Pretest/Posttest	Developed	Not specified	2018	Belgium

								rhythm were excluded					
Contact-free screening of atrial fibrillation by a smartphone using facial pulsatile photoplethysmographic signals	Yan, B.P. et al.	Atrial fibrillation	Cardiology	Screening	Smartphone application	Smartphone	iPhone Operating System	Patients admitted to the cardiology ward of the hospital for clinical reasons	Experimental	Developed	Not specified	2018	Multinational
Diagnostic Performance of a Smartphone-Based Photoplethysmographic Application for Atrial Fibrillation Screening in a Primary Care Setting.	Chan, P. et al.	Atrial fibrillation	Cardiology	Screening	Smartphone application	Smartphone	iPhone Operating System	Patients with hypertension, with diabetes mellitus, and/or aged ≥65 years	Pretest/Posttest	Developed	Not specified	2016	Hong Kong
Performance and Usability of a Novel Smartphone Application for Atrial Fibrillation Detection in an Ambulatory Population Referred for Cardiac Monitoring	Sardana, M. et al.	Atrial fibrillation	Cardiology	Diagnosing/ Monitoring	Smartphone application	Smartphone	iPhone Operating System	Ambulatory patients, referred for cardiac event monitoring for cryptogenic stroke or suspected AF	Experimental	Not specified	Not specified	2016	United States
Smartwatch Algorithm for Automated Detection of Atrial Fibrillation	Bumgarner, J.M. et al.	Atrial fibrillation	Cardiology	Diagnosis	Smartwatch algorithm	Apple smartwatch	iPhone Operating System	Consecutive patients with a diagnosis of AF who presented for scheduled elective CV with or without a planned transesophageal echocardiogram	Experimental	Prototype	Not specified	2018	United States
A novel application for the detection of an irregular pulse using an iPhone 4S in patients with atrial fibrillation	McManus, D.D. et al.	Atrial fibrillation	Cardiology	Diagnosis	Smartphone application	Smartphone	iPhone Operating System	Adults with persistent AFib	Experimental	Prototype	Not specified	2013	United States
Atrial fibrillation detection using an iPhone 4S	Lee, J. et al.	Atrial fibrillation	Cardiology	Diagnosis	Smartphone application	Smartphone	iPhone Operating System	AF subjects undergoing electrical cardioversion	Experimental	Prototype	Not specified	2013	United States
Arrhythmia discrimination using a smart phone.	Chong, J. W. et al.	Arryttmia	Cardiology	Diagnosis	Smartphone application	Smartphone	iPhone Operating System	Subjects with PVC and AC subjects as well as AF pre- and post- electrical cardioversion	Experimental	Prototype	Not specified	2013	United States
PULSE-SMART: Pulse-Based Arrhythmia Discrimination Using a Novel Smartphone Application	DAVID D. McMANUS, JO WOON CHONG, APURV SONI	Arrythmia	Cardiology	Diagnosing	Mobile application	Smartphone	iPhone Operating System	Patients with both benignant and malignant causes of irregular pulse.	Pretest/Posttest	Validation	Not specified	2016	United States
The RITMIA+ Smartphone App for Automated Detection of Atrial Fibrillation: Accuracy in Consecutive Patients Undergoing Elective Electrical Cardioversion	Claudio Reverberi	Atrial fibrillation	Cardiology	Monitoring	RITMIA App	Smartphone	iPhone Operating System or Android	Patients with atrial fibrillation	Observational	Developed	Not specified	2019	Italy
Study on Real-time Monitoring Technique for Cardiac Arrhythmia Based on Smartphone	Weng J, et al.	Cardiac arrhythmia	Cardiology	Diagnosis/ Monitoring	Software for an ECG monitoring system on a smartphone platform	Smartphone	Microsoft and Blackberry	NA	Technical testing	Not specified	Not specified	2012	China
Novel Methods of Faster Cardiovascular Diagnosis in Wireless Telecardiology	Sufi, F. et al.	Cardiovascular abnormalities (e.g. tachycardia,	Cardiology	Diagnosis/ Monitoring	Generic ECG compression algorithm suitable for	Mobile phones	MIDlet implementation of J2ME using	None	Technical testing	Prototype	Not specified	2009	Australia

			bradycardia, arrythmia, Bundle Branch Block, Premature Ventricular Contraction, Wolff-Parkinson-White syndrome, etc.)			SMS/MMS compatile wireless telecardiology		Netbeans 5.5 IDE [22]					
	Excellent Symptom Rhythm Correlation in Patients with Palpitations Using A Novel Smartphone Based Event Recorder	William George Newhama, Muzahir Hassan Tayebjee	Palpitations	Cardiology	Diagnosis	Mobile application	Smartphone	Not specified	Patients with palpitations	Experimental	Available	At £74.99	2017	Unite Kingdo m
	Continuous heart rate monitoring for automatic detection of atrial fibrillation with novel bio-sensing technology	Hochstadt, A. et al.	Atrial fibrillation	Cardiology	Diagnosis	PPG Wrist-watch sensor	Custom-made wearable photo-plethysmograph [PPG] wrist-watch sensor	Not specified	Patients undergoing elective cardioversion of AF	Experimental	Developed	Not specified	2018	Israel
	Smart watches for heart rate assessment in atrial arrhythmias	Koshy, A.N. et al.	Arrythmias	Cardiology	Monitoring	Smartwatch software	FitBit (FB), Apple Watch (AW)	Not specified	Hospitalized patients	Pretest/Postest	Developed	Not specified	2018	Australi a
	Detection of premature ventricular contractions using the RR-interval signal: a simple algorithm for mobile devices	Cuesta, P. et al.	Premature ventricular contractions	Cardiology	Diagnosis	Algorithm for mobile devices	Mobile devices	Not specified	None	Technical testing	Developed	Free appication	2014	Spain
	Assessing the accuracy of an automated atrial fibrillation detection algorithm using smartphone technology: The iREAD Study	William, A.D. et al.	Atrial fibrillation	Cardiology	Diagnosis	Smartphone-coupled monitor	Smartphone coupled monitor	Not specified	Consecutive patients with AF admitted for antiarrhythmic drug initiation	Experimental	Developed	Not specified	2018	United States
	Diagnostic utility of smartphone ECG technology in the initial investigation of palpitations	Dimarco, A.D. et al.	Palpitations	Cardiology	Diagnosis	Smartphone application	Smartphones	Not specified	Patients that experienced palpitations less than daily and had access to, and the ability to use, a smartphone.	Experimental	Developed	Not specified	2019	United Kingdo m
	Trial design of the WATCH AF trial - SmartWATCHes for detection of atrial fibrillation	Doerr, M. et al.	Atrial fibrillation	Cardiology	Diagnosis	Smartwatch software	Smartwatch (Samsung) and a wristband (Wavelet health)	Not specified	Subjects of legal age	Experimental	Developed	Not specified	2017	German y
	Automated Real-Time Atrial Fibrillation Detection on a Wearable Wireless Sensor Platform	Francisco Rincon	Atrial fibrillation	Cardiology	Monitoring	Wearable device	Wearable wireless sensor	Not specified	None	Technical testing	Developed	Not specified	2012	Multinat ional
	Atrial Fibrillation Detection Using a Novel Cardiac Ambulatory Monitor Based on Photo-Plethysmography at the Wrist	Bonomi, A.G. et al.	Atrial fibrillation	Cardiology	Diagnosis/ Monitoring	Wrist-wearable device	Wrist-wearable device	Not specified	AF patients undergoing electrical cardioversion (ECV) and AF patients that were prescribed for 24 hours ECG Holter in outpatient settings (HOL).	Experimental	Developed	Not specified	2018	Netherla nds
Parkinsonian Disorder (N=18)	Parkinson's disease and essential tremor classification on mobile device	Alan Michael Woods et al	Parkinson Disease and Essential Tremor	Neurology	Diagnosis/ Screening	App on smartphone	Smartphone	Android	Fourteen participants diagnosed with PD and eighteen participants with ET undertook our study	Technical testing	Developed	Not specified	2014	New Zealand

Gait monitoring for early neurological disorder detection using sensors in a smartphone: Validation and a case study of parkinsonism	Raknim, P., & Lan, K.	Parkinsonism	Neurology	Diagnosis/ Monitoring	Smartphone application	Smartphone	Android	Senior patients	Experimental	Developed	Not specified	2016	Taiwan
Parkinson's disease hand tremor detection system for mobile application	Fraiwan, L. et al.	Parkinson's Disease	Neurology	Diagnosis	Smartphone built-in accelerometer	Smartphone	Android	PD patients and healthy controls	Experimental	Developed	Not specified	2016	United Arab Emirate s
Predicting motor, cognitive & functional impairment in Parkinson's	Christine Lo, Siddharth Arora, Fahd Baig et al	Parkinson's Disease	Neurology	Screening	Smartphone recordings	Smartphone	Android	Participants being ascribed a probability of Parkinson's of at least 90% by trained researchers at their latest clinic assessment.	Experimental	Developed	Not specified	2019	United Kingdo m
A Smartphone Application for Automated Decision Support in Cognitive Task Based Evaluation of Central Nervous System Motor Disorders.	Lauraitis, A. et al.	Huntington, Alzheimer or Parkinson diseases	Neurology	Diagnosis	Smartphone application	Tablet - Samsung Galaxy Tab and Smartphone - Samsung S7	Android	None	Technical testing	Developed	Free on Google Play Store	2019	Lithuani a
Detecting and monitoring the symptoms of Parkinson's disease using smartphones: A pilot study	Arora, S. et al.	Parkinson's Disease	Neurology	Diagnosis/ Monitoring	Smartphone application	Smartphone	Android	Individuals with PD diagnosed clinically by a movement disorder specialist and control participants	Experimental	Pilot	Not specified	2015	Multinat ional
Detection of Motor Impairment in Parkinson's Disease Via Mobile Touchscreen Typing.	Arroyo-Gallego, T. et al.	Parkinson's Disease	Neurology	Diagnosis	Smartphone typing test	Smartphone	Android	24 people diagnosed with Parkinson's and 27 healthy controls	Experimental	Pilot study	Not specified	2017	Multinat ional
A Mobile Cloud-Based Parkinson's Disease Assessment System for Home-Based Monitoring	Pan, D. et al.	Parkinson's Disease	Neurology	Monitoring	Smartphone application	Smartphone	Android	PD Patients	Experimental	Prototype	Not specified	2015	United States
Parkinson's Disease Classification of mPower Walking Activity Participants	Benjamin Pittman	Parkinson´s Disease	Neurology	Monitoring	Smartphone application	Smartphone	iPhone Operating System	Volunteers	Experimental	Developed	Not specified	2018	United States
A Smartphone-Based Tool for Assessing Parkinsonian Hand Tremor	Kostikis, N. et al.	Parkinson's Disease	Neurology	Diagnosis	Smartphone application	Smartphone	iPhone Operating System	PD patients and age-matched healthy volunteers	Experimental	Developed	Not specified	2015	Greece
Validating an iPhone Operating System-based rhythmic auditory cueing evaluation (iRACE) for Parkinson's disease	Zhu, S. et al.	Parkinson's Disease	Neurology	Monitoring	Smartphone application	iPhone or iPod Touch	iPhone Operating System	PD Patients	Experimental	Developed	Not specified	2014	Singapo re
A validated smartphone-based assessment of gait and gait variability in parkinson's disease	Ellis, R.J. et al.	Parkinson's Disease	Neurology	Monitoring	Smartphone application	iPod Touch	iPhone Operating System	PD patients and healthy controls	Experimental	Developed	Not specified	2015	Singapo re
Parkinson's Disease Classification of mPower Walking Activity Participants	Pittman, B. et al.	Parkinson's Disease	Neurology	Screening/ Diagnosis	Smartphone application	Smartphone	iPhone Operating System	None	Technical testing	In development	Not specified	2018	United States
Implementation of an iPhone for characterizing Parkinson's disease tremor through a wireless	Robert LeMoyne et al	Parkinson's Disease	Neurology	Monitoring	Iphone wireless accelerometer application	Smartphone	iPhone Operating System	One Parkinson disease diagnosed patient and a control individual	Experimental	Not specified	Not specified	2010	United States

	accelerometer application													
	Robust Detection of Parkinson's Disease Using Harvested Smartphone Voice Data: A Telemedicine Approach	Singh, S. and Xu, W.	Parkinson's Disease	Neurology	Diagnosis	Smartphone application	Smartphone	iPhone Operating System	Unique participants	Experimental	Developed	Not specified	2019	United States
	Computerized spiral analysis using the iPad	Sisti, J.A. et al.	Movement Disorders (essential tremor, Parkinson´s Disease, dystonia, etc.)	Neurology	Monitoring	CSA Ipad Application	Tablet	iPhone Operating System	Healthy controls and patients with moving disorders	Experimental	Developed	Not specified	2016	United States
	Non-Contact Human Gait Identification throughIR-UWB Edge Based Monitoring Sensor	Soumya Prakash Rana	Parkinson's Disease	Neurology	Monitoring	UWB pulsed radar	UWB P410 radar module	Not specified	Test subjects	Technical testing	Developed	Not specified	2019	England
	Parkinson's disease and essential tremor classification on mobile device	Woods, A.M. et al.	Parkinson's Disease	Neurology	Diagnosis	Smartphone application	Smartphone	Not specified	Participants diagnosed with PD and with ET	Experimental	Prototype	Not specified	2014	New Zealand
Retinal Diseases (N=14)	An intelligent mobile based decision support system for retinal disease diagnosis	Bourouis, A. et al.	Retinal disease	Ophthalmology	Diagnosis	Smartphone based intelligent system	Smartphone	Android	Test images	Experimental	Developed	Not specified	2014	Multinational
	Decision Support System for Detection of Diabetic Retinopathy Using Smartphones	Prasanna, P. et al.	Diabetic Retinopathy	Ophthalmology	Screening	Smartphone application	Smartphone	Android	None	Technical testing	Developed	Not specified	2013	United States
	Enduring eye care with smartphones aiding real time diagnosis.	Sudhakar, M.S. & Bhoopathy Bagan K.	Retinal pathologies	Ophthalmology	Diagnosis	Add-on for smartphones	Smartphone	Android	None	Technical testing	Developed	Not specified	2014	India
	Wide-field smartphone fundus video camera based on miniaturized indirect ophthalmoscopy	Toslak, D. et al	Retinal disease	Ophthalmology	Screening	Smartphone application	Smartphone	Android	Subjects without eye diseases	Technical testing	Proof of principle	Not specified	2018	Multinational
	Smartphone-Based Accurate Analysis of Retinal Vasculature towards Point-of-Care Diagnostics	Xiayu Xu et al	Diabetic retinopathy, various diseases	Ophthalmology	Diagnosis	Android app	Smartphone	Android	40 funduscopic from a data base and 10 healthy patients	Experimental	Developed	Not specified	2016	China
	Retinal imaging with smartphone.	Ademola-Popoola, D., Olatunji, V.	Retinal diseases	Ophthalmology	Diagnosis	Smartphone camera for retinal imaging	Smartphone	Blackberry	Patients aged between 15 months and 61 years with various diagnosis.	Experimental	Tested in clinical practice	Not specified	2017	Nigeria
	Automatic diabetic retinopathy diagnosis using adjustable ophthalmoscope and multi-scale line operator	Qu, M. et al.	Diabetic retinopathy	Ophthalmology	Diagnosis/ Screening	Smartphone ophthalmoscope	Smartphone	iPhone Operating System	Patients after pupil dilation	Pretest/Postest	In development	Not specified	2017	China
	MII RetCam assisted smartphone-based fundus imaging for retinopathy of prematurity	Lekha, T. et al.	Retinopathy of prematurity	Ophthalmology	Diagnosis/ Monitoring	Smartphone add-on	Smartphone	iPhone Operating System	All the preterm babies subjected to MSFI as part of ROP screening from September 2017 to November 2018	Observational	Developed	MII RetCam device costs USD 380/-	2019	India
	Mobile phones for retinopathy of prematurity screening in Lagos, Nigeria, sub-Saharan Africa	Tunji S. Oluleye , Adekunle Rotimi-Samuel , Adetunji Adenekan	Retinopathy of prematurity	Ophthalmology	Screening	Mobile application	Smartphone	iPhone Operating System	Preterm infants with birthweight of less than 1.5 kg or gestational age of less than 32 weeks	Technical testing	Available in App Store	Not specified	2016	Nigeria

	A Smartphone-Based Tool for Rapid, Portable, and Automated Wide-Field Retinal Imaging.	Kim, T. et al.	Retinal diseases	Ophthalmology	Screening	Smartphone application	Smartphone	iPhone Operating System	Subjects from the University of Michigan Kellogg Eye Center Retina Clinic	Experimental	Developed	Not specified	2018	United States
	A mobile phone-based retinal camera for portable wide field imaging	Maamari, R. et al.	Retinal diseases	Ophthalmology	Screening	Mobile phone-based retinal camera	Smartphone	iPhone Operating System	None	Technical testing	Prototype	Estimated cost $59.64	2013	United States
	3D Printed Smartphone Indirect Lens Adapter for Rapid, High Quality Retinal Imaging	Myung, D. et al.	Retinal diseases	Ophthalmology	Diagnosis	Smartphone application	Smartphone	iPhone Operating System	None	Technical testing	Prototype	Not specified	2014	United States
	Smartphone-based fundus camera device (MII Ret Cam) and technique with ability to image peripheral retina	Sharma, A. et al.	Retinal pathologies	Ophthalmology	Diagnosis	Smartphone (with built in flashlight and camera)	Smartphone (with built in flashlight and camera)	Not specified	First year residents of Opthalmology	Experimental	Developed	Not specified	2015	India
	Automated diabetic retinopathy detection in smartphone-based fundus photography using artificial intelligence	Ramachandra n Rajalakshmi1	Diabetic retinopathy	Ophthalmology	Screening	Artificial Intelligence	Smartphone	Not specified	Patients	Experimental	Developed	Not specified	2018	India
Hearing loss (N=13)	Clinical Validity of hearScreen™ Smartphone Hearing Screening for School Children	Mahomed-Asmail, Faheem, Swanepoel, De Wet, Eikelboom, Robert H, Myburgh, Hermanus C Hall, James 3rd	Hearing loss	Otorrhinolaryn gology	Screening	Mobile application	Smartphone	Android	School-age children from grade 1 to 3 with an average age of 8 years (±1.1 SD; range 5 to 12 years) were recruited from five public government schools in underserved regions of the Gauteng Province, South Africa.	Pretest/Postest	Available	Not specified	2015	South Africa
	Hearing Tests Based on Biologically Calibrated Mobile Devices: Comparison With Pure-Tone Audiometry.	Marcin Masalski, MD, PhD ; Tomasz Grysiński, PhD (Eng) ; Tomasz Kręcicki, PhD, MD	Hearing loss	Otorrhinolaryn gology	Monitoring	Mobile application	Smartphone	Android	Patients of Otolaryngology Clinic	Pretest/Postest	Available	Free	2018	Poland
	Smartphone-Based Hearing Screening at Primary Health Care Clinics	Christine Louw, De Wet Swanepoel, Robert H. Eikelboom, et al	Hearing loss	Otorrhinolaryn gology	Screening	Mobile application	Smartphone	Android	Patients from two Primary Health Care Center in Twsahne Province, South Africa.	Experimental	Available	Free	2016	South Africa
	Extended High-Frequency Smartphone Audiometry: Validity and Reliability.	Bornman, M. et al.	Age-related hearing loss, noise-induced hearing loss (NIHL) and ototoxicity	Otorrhinolaryn gology	Screening	Smartphone application	Samsung Galaxy Trend Neo	Android	Participants were recruited from adults attending the Audiology Department at Dr. George Mukhari Hospital, GaRankuwa, South Africa and from the University of Pretoria	Experimental	Developed	Not specified	2019	Multinat ional
	Community-based hearing screening for young children using an mHealth service-delivery model	Hussein, S. et al.	Hearing loss	Otorrhinolaryn gology	Screening	Smartphone-based hearding screening program.	Smartphone	Android	Children (3-6 years old)	Experimental	Developed	Not specified	2018	South Africa

	Evaluation of the Hearing Test Pro Application as a Screening Tool for Hearing Loss Assessment.	Aremu, Shuaib Kayode	Hearing loss	Otorrhinolaryngology	Screening	Smartphone app as screening tool for hearing loss	Smartphone	Android	Adult android-phone users	Pretest/Postest	Available	Not specified	2018	Nigeria
	Tablet-Based Hearing Screening Test	Alessandra G. Samelli	Hearing loss	Otorrhinolaryngology	Screening	Tablet-based software	Apple Ipad and Dell Notebook PC	iPhone Operating System	Patients with diverse types of hearing loss	Observational	Developed	Not specified	2017	Brazil
	Implementation of uHear™ - an iPhone Operating System-based application to screen for hearing loss - in older patients with cancer undergoing a comprehensive geriatric assessment	Michelle Lyckea , Tom Boterbergb , Evi Martens et al	Presbycusis	Otorrhinolaryngology	Screening	Mobile application	iPod, iPhone, iPad	iPhone Operating System	Older patients with cancer at the radiotherapy and oncology departments of the General Hospital Groeninge (Kortrijk, Belgium) from December 2014 till June 2015	Pretest/Postest	Available	Not specified	2016	Belgium
	Application-Based Hearing Screening in the Elderly Population	Leonid Livshitz, Reem Ghanayim, Carmi Kraus et al	Presbycusis (Hearing loss)	Otorrhinolaryngology	Screening	Mobile application	Tablet	iPhone Operating System	Patients 65 years of age or older hospitalized for any reason in an internal medicine department	Experimental	Available	Free	2017	United States
	Initial assessment of hearing loss using a mobile application for audiological evaluation	Derin, S. et al	Hearing loss	Otorrhinolaryngology	Diagnosis	Mobile application	Smartphone	iPhone Operating System	Patients with hearing loss	Pretest/Posttest	Developed	Not specified	2016	Turkey
	Smartphone-based audiometric test for screening hearing loss in the elderly.	Abu-Ghanem, S. et al.	Hearing loss	Otorrhinolaryngology	Screening	Smartphone app as screeing tool for hearing loss	Smartphone - iPhone and Tablet - iPod, iPad	iPhone Operating System	Subjects aged 84.4 ± 6.73 years (mean ± SD) were recruited.	Pretest/Posttest	Available	Free	2015	Israel
	Hearing loss in the developing world: evaluating the iPhone mobile device as a screening tool.	Peer, S. & Fagan, J.	Hearing Loss (High risk: Presbycusis, HIV and TB Therapy, Chemotherapy)	Otorrhinolaryngology	Screening	Smartphone application	Smartphone	iPhone Operating System	Participants recruited from the Otolaryngology Clinic, Groote Schuur Hospital, Cape Town, South Africa	Pretest/Posttest	Developed	Free	2014	South Africa
	Hearing tests are just child's play: the sound scouts' game for children entering school.	Dillon, H. et al.	Hearing loss	Otorrhinolaryngology	Screening	Tablet-based hearing test	Tablet	iPhone Operating System or Android	Children (n=213) and adults (n=20) screened to have hearing thresholds of 20dB or better.	Experimental	Developed	Not specified	2018	United Kingdom
Melanoma and Skin Cancer (N=11)	Melanoma and other skin lesion detection using smart handheld devices.	G Zouridakis et al	Melanoma	Dermatology	Diagnosis	Mobile application	Smartphone	iPhone Operating System	None	Technical testing	Not specified	Not specified	2014	Spain
	Cell phone usefulness to improve the skin cancer screening: preliminary results and critical analysis of mobile app development	Goulart, C.E. et al.	Skin Cancer	Dermatology	Screening	Smartphone application	Smartphone	Android	Individuals monitored by routine skin cancer screening by the Cancer Prevention Department at Barretos Cancer Hospital during 2016	Pretest/Posttest	Developed	Not specified	2019	Brazil
	Early melanoma diagnosis with mobile imaging	Do, T-T. et al	Melanoma	Dermatology	Diagnosis	Smartphone application	Smartphone (Samsung Galaxy S4)	Android	NA	Technical testing	Prototype	Not specified	2014	Singapore
	Skin Cancer Diagnostics with an All-Inclusive Smartphone Application	Kalwa, U. et al.	Skin Cancer	Dermatology	Diagnosis	Smartphone application	Smartphone (Samsung S6)	Android	Images with Synthetic Minority Over-sampling Technique (SMOTE) and without SMOTE	Experimental	Prototype	Not specified	2019	United Kingdom
	Mobile teledermoscopy-	Borve, Alexander	Skin cancer	Dermatology	Screening	Mobile app and customised dermoscope	Smartphone	iPhone Operating System	Patients with one or more suspicious skin	Experimental	Not specified	Not specified	2013	Sweden

	there's an app for that!								lesions deemed to need a biopsy or excision within a 16 week period.					
	SkinScan©: A Portable Library for Melanoma Detection on Handheld Devices	Wadhawan, T. et al	Melanoma	Dermatology	Screening	Smartphone application	Smartphone	iPhone Operating System	Not specified	Technical testing	Proof of principle	Not specified	2011	United States
	Implementation of the 7-point checklist for melanoma detection on smart handheld devices	Wadhawan, T. et al	Melanoma	Dermatology	Diagnosis	Smartphone application	Smartphone and iPad	iPhone Operating System	Not specified	Technical testing	Proof of principle	Not specified	2011	United States
	SkinScan©: a portable library for melanoma detection on handheld devices	Wadhawan, T. et al.	Melanoma	Dermatology	Diagnosis	Smartphone application	iPhone 4, iPod, iPad and Android-based devices	iPhone Operating System and Android	Artifact free images	Experimental	Developed	Not specified	2011	United States
	Dermatologist-level classification of skin cancer with deep neural networks.	Esteva, A. et al.	Skin cancer	Dermatology	Screening/ Diagnosis	Single convolutional neural network algorithm	Smartphone	Not specified	Clinical images including dermoscopy images	Pretest/Posttes t	Developed	Not specified	2017	United States
	Mobile teledermatology for skin tumour screening: diagnostic accuracy of clinical and dermoscopic image tele-evaluation using cellular phones	Kroemer, S. et al.	Skin tumour	Dermatology	Screening	Telemedicine system	Nokia N73	Not specified	Patients from the general outpatient clinic at the Department of Dermatology, Medical University of Graz, Graz, Austria	Experimental	Developed	Not specified	2011	Austria
	Melanoma Screening with Cellular Phones	Massone, C. et al	Melanoma	Dermatology	Screening	Smartphone application	Sony Ericsson k750i	Not specified	Patients that attended the Pigmented Skin Lesions Clinic of the Department of Dermatology, Medical University of Graz during two routine working days.	Experimental	Proof of principle	Not specified	2007	Austria
Diabetes (N=10)	Interactive diary for diabetes: a useful and easy-to-use new telemedicine system to support the decision-making process in type 1 diabetes	Rossi. M. et al.	Diabetes	Endocrinology	Monitoring	Telemedicine system	Mobile phones	Not specified	Type 1 Diabetes patients	Experimental	Developed	Not more than 1.5 Euros	2009	Multinat ional
	Mobile communication using a mobile phone with a glucometer for glucose control in Type 2 patients with diabetes: as effective as an Internet-based glucose monitoring system	Cho, J-H. et al.	Diabetes	Endocrinology	Monitoring	Mobile phone	Mobile Health-care diabetes phone; LG-KP8400	Not specified	Type 2 diabetes patients	Experimental	Developed	Not specified	2009	Republi c of Korea
	Design of an mHealth app for the self-management of adolescent type 1 diabetes: a pilot study	Cafazzo, J. A. et al.	Diabetes	Endocrinology	Monitoring	Smartphone application	iPhone 4 or iPod Touch	iPhone Operating System	Adolescents with type 1 diabetes	Experimental	Pilot	Not specified	2012	Canada
	Diabetes self-management smartphone application for adults with type 1 diabetes: randomized controlled trial	Kirwan, M. et al.	Diabetes	Endocrinology	Monitoirng	Smartphone application	Smartphone	iPhone Operating System	Adult patients with type 1 Diabetes	Experimental	Developed	Free application	2013	Australi a
	Re-usable electrochemical glucose sensors	Bandodkar, A. et al.	Diabetes	Endocrinology	Monitoring	Smartphone-based reusable glucose meter	Smertphone	Android	NA	Technical testing	Prototype	Not specified	2018	United States

	integrated into a smartphone platform.													
	Evaluation of a mobile phone telemonitoring system for glycaemic control in patients with diabetes	Istepanian, R. et al.	Diabetes	Endocrinology	Monitoring	Mobile phone-based system	Motorola A-100 mobile phone	Android	Patients with complicated diabetes	Experimental	Not specified	Not specified	2009	United Kingdo m
	Ultrabright Polymer-Dot Transducer Enabled Wireless Glucose Monitoring via a Smartphone	Sun, K. et al.	Diabetes	Endocrinology	Monitoring	Smartphone application	Smartphone-Huawei Mate 9	Android	Balb/c nude mice (Vital River Laboratories, Beijing, China). 8-week-old female mice	Experimental	In vitro and in vivo studies	Not specified	2018	China
	Real time monitoring of glucose in whole blood by smartphone	Erenas, M. et al.	Diabetes	Endocrinology	Monitoring	Combined thread-paper microfluidic device	Sony DSC-HX300 digital camera, a Samsung Galaxy S5 smartphone, a Samsung Galaxy Tab A tablet, and a Motorola Moto G4 Play smartphone	Android	None	Technical testing	Developed	Not specified	2019	Multinat ional
	Smartphone based non-invasive salivary glucose biPhone Operating Systemensor	Soni, A. & Jha, S.K.	Diabetes	Endocrinology	Diagnosis/ Screening	Smartphone application	Smartphone	Android	Subjects between age group 20-80 years at Outpatient Department of Indian Institute of Technology Delhi hospital, New Delhi	Experimental	Developed	Not specified	2017	India
	Non-invasive blood glucose monitor based on spectroscopy using a smartphone.	Dantu, V., Vempati, J., Srivilliputhur, S.	Diabetes	Endocrinology	Monitoring	Non invasive blood glucose monitor	Smartphone	Android	Human subjects who drank Cola beverage of 50g sugar	Pretest/Posttes t	Developed	Not specified	2014	United States
Anemia (N=7)	Cost-effective and rapid blood analysis on a cellphone	Hongying Zhu, Ikbal Sencan, Justin Wong et al	Anemia	Hematology	Diagnosis	Mobile application	Smartphone	Android	Anonymous human whole blood samples obtained from UCLA Blood and Platelet Center	Experimental	Developed	Not specified	2013	United States
	3D printed auto-mixing chip enables rapid smartphone diagnosis of anemia	Plevniak, K. et al.	Anemia	Hematology	Diagnosis	Smartphone application	Smartphone	Android	Patients and healthy donors aged 25-39 years and resident of the site zone	Experimental	In development	The overall cost per test is 50 cents.	2016	United States
	Histogram analysis for smartphone-based rapid hematocrit determination	Jalal, U., Kim, S., Shim, J.	Anemia	Hematology	Diagnosis	Lab-on-a-chip platform including a disposable microfluidic device and a smartphone app	Smartphone	Android	Healthy adult blood donor	Technical testing	Developed	US $300 (includes smartphone cost)	2017	South Korea
	Smartphone app for non-invasive detection of anemia using only patient-sourced photos	Robert G. Mannino, David R. Myers, Erika A. Tybursk et al	Anemia	Hematology	Screening	Mobile application	Smartphone	Android and iPhone Operating System	Patients with a variety of anemia diagnosis mixed with healthy subjects	Experimental	Developed	Not specified	2018	United States
	Detection and quantification of subtle changes in red blood cell density using a cell phone.	Felton, E. et al.	Anemia	Hematology	Diagnosis	Smartphone add-on	Smartphone	iPhone Operating System	Multiple donors	Technical testing	Developed	Approx. $45.75 (iPhone device parts)	2016	United States
	Development and validation of a noncontact spectroscopic device for hemoglobin estimation at point-of-care	Sarkar, P.K. et al.	Anemia	Hematology	Screening	Software application running on a tablet computer	Tablet computer	Not specified	Patients of all ages, sex, and skin color	Pretest/Posttes t	In development	Not specified	2017	India

	Optofluidic fluorescent imaging cytometry on a cell phone.	Hongying Zhu et al	Anemia	Clinical Lab	Diagnosis/ Monitoring	Integraton of flow cytometry and optical microscopy on a cel phone for rapid blood analysis	Sony Ericsson UIOI Aino Samsung Galaxy SII (Smartphone)	Android and iPhone Operating System	Random 12 patients for blood analysis	Pretest/Posttest	Developed	Simple lens (cost, <3 USD/piece), a plastic color filter (cost, <0.1 USD/piece), two lightemitting diodes (LEDs) (cost, ~0.3 USD/piece), and batteries (cost, ~0.5 USD/piece), which make it rather cost-effective.	2011	United States
Visual Impairment (N=7)	Validation of dynamic random dot stereotests in pediatric vision screening.	Budai, A. et al.	Amblyopia, Anisometropia, Convergent strabismus, and Hyperopia	Ophthalmology	Screening	Tablet-based visual acuity test	Tablet	Android	Children with diagnoses of amblyopia, anisometropia, conver- gent strabismus, and hyperopia, as well as healthy subjects.	Pretest/Posttest	Not specified	Not specified	2019	Hungary
	Clinical Validation of a Smartphone-Based Adapter for Optic Disc Imaging in Kenya.	Bastawrous, Andrew	Visual impairment	Ophthalmology	Diagnosis	Smartphone application	Smartphone	Android	Adults 55 years and older	Pretest/Posttest	Prototype	Not specified	2016	United Kingdom
	Automated Measurement of Visual Acuity in Pediatric Ophthalmic Patients Using Principles of Game Design and Tablet Computers.	Aslam, T. et al.	Visual impairment: Amblyopia, Cataract, Corneal opacity, Retinal disease	Ophthalmology	Screening	Software application running on a tablet device	Tablet	Android	Patients from a pediatric ophthalmology outpatient clinic	Pretest/Posttest	Tested in clinical practice	Not specified	2016	United Kingdom
	Performance of an iPad Application to Detect Moderate and Advanced Visual Field Loss in Nepal.	Johnson, C. et al.	Visual field loss	Ophthalmology	Screening	Tablet application	Tablet	iPhone Operating System	Subjects: normal (NL), with glaucoma (GL) and with diabetic retinopathy (DR) at Tilganga Institute of Ophthalmology, Kathmandu, Nepal.	Pretest/Posttest	Developed	Free	2017	Multinational
	Normative values for a tablet computer-based application to assess chromatic contrast sensitivity.	Bodduluri, L. et al.	Color vision deficiencies (could relate to ocular conditions such as diabetic retinopathy, age-related macular degeneration and glaucoma)	Ophthalmology	Diagnosis	Tablet computer - based games	Tablet	iPhone Operating System	Healthy control participants with a VA of 6/6 or better, measured with a Bailey Lovie LogMAR VA chart	Pretest/Posttest	Developed	Not specified	2017	Australia
	Evaluation of a smartphone photoscreening app to detect refractive amblyopia risk factors in children aged 1-6 years.	Arnold, R. et al.	Refractive amblyopia risk factors	Ophthalmology	Screening	Photoscreening tool	Smartphone	iPhone Operating System	Children aged 1-6 years	Pretest/Posttest	Food and Drug Administration Approved device	Not specified	2018	United States
	The Efficacy of a Novel Mobile Phone Application for Goldmann Ptosis	Robi N. Maamari, Michael V. D'Ambrosio,	Superior visual field obstruction	Ophthalmology	Screening	Mobile application	Smartphone	iPhone Operating System	Ophthalmology board certified and fellowship-trained oculoplastic	Technical testing	In development	Not specified	2014	United States

Visual Field Interpretation	Jeffrey M. Joseph et al							surgeons who routinely use Goldmann VF testing in their practice					

Abbreviations: ECG: Electrocardiogram; USA: United States of America

Supplementary Table 4. Studies with most advanced stage of development

Title	**Authors**	**Name of the Technology**	**Disease RF**	**Clinical Domain**	**Aim**	**Type of Intervention**	**Mobile**	**OS**	**Study Population**	**Methods**	**Evaluation Values**	**Stage of Development**	**Cost**	**Year**	**Author Affiliation**
A smartphone application to support recovery from alcoholism: a randomized clinical trial	Gustafson, D.H. et al.	Addiction–Comprehensive Health Enhancement Support System (A-CHESS)	Alcoholism	Psychiatry	Monitoring	Smartphone application	Smartphone	Not specified	Patients who met the criteria for DSM-IV alcohol dependence when they entered residential treatment	Experimental	Variability measures	Developed	8 months of A-CHESS cost about $597 per patient	2014	United States
The utility of hand-held mobile spirometer technology in a resource-constrained setting.	Du Plessis, E. et al.	Air-Smart Spirometer	Chronic respiratory diseases	Pneumology	Screening	Smartphone application	Smartphone	Not specified	Consecutive patients and healthy volunteers	Experimental	Measures of diagnostic accuracy	Developed	Not specified	2019	South Africa
Screening for atrial fibrillation in 13 122 Hong Kong citizens with smartphone electrocardiogram	Chan, N. & Choy, C.	AliveCor	Atrial fibrillation	Cardiology	Screening	Smartphone-based wireless single-lead ECG (SL-ECG)	Smartphone	iPhone Operating System	Hong Kong citizens	Experimental	Measures of diagnostic accuracy	Developed	Not specified	2016	China
SPEAR Trial: Smartphone Pediatric ElectrocARdiogram Trial	Nguyen, H.H. et al.	AliveCor	ECG Monitoring	Cardiology	Diagnosis	Smartphone application	Smartphone	iPhone Operating System	Pediatric patients with age 18 years or younger, with paroxysmal arrhythmia	Pretest/Posttest	Not specified	Developed	Not specified	2015	United States
Diagnostic utility of smartphone ECG technology in the initial investigation of palpitations	Dimarco, A.D. et al.	AliveCor	Palpitations	Cardiology	Diagnosis	Smartphone application	Smartphone	Not specified	Patients that experienced palpitations less than daily and had access to, and the ability to use, a smartphone.	Experimental	Not specified	Developed	Not specified	2019	United Kingdom
Wireless Smartphone ECG Enables Large-Scale Screening in Diverse Populations.	Haberman, Z. et al.	AliveCor ECG	ECG Monitoring	Cardiology	Screening	Wireless Smartphone ECG	Smartphone and tablet	iPhone Operating System and Android	University of Southern California (USC) Division I Athletes, asymptomatic USC students and ambulatory USC cardiology clinic patients	Experimental	Measures of diagnostic accuracy	Developed	Not specified	2015	United States
Self-monitoring for atrial fibrillation recurrence in the discharge period post-cardiac surgery using an iPhone electrocardiogram	Lowres, N. et al.	AliveCor Heart monitor	Atrial fibrillation	Cardiology	Monitoring	Smartphone-based wireless single-lead ECG (SL-ECG)	Smartphone	iPhone Operating System	Patients with POAF following cardiac surgery	Observational	Not specified	Developed	Not specified	2016	Australia

Feasibility and cost-effectiveness of stroke prevention through community screening for atrial fibrillation using iPhone ECG in pharmacies. The SEARCH-AF study	Lowres, N. et al.	AliveCor Heart Monitor	Atrial fibrillation	Cardiology	Screening	Smartphone application	Smartphone	iPhone Operating System	Pharmacy customers aged ≥65 years	Observational	Measures of diagnostic accuracy	Developed	Not specified	2014	Multinational
iPhone ECG application for community screening to detect silent atrial fibrillation: a novel technology to prevent stroke	Lau, J. et al.	AliveCor iPhone ECG	Atrial fibrillation	Cardiology	Screening	Smartphone application	Smartphone	iPhone Operating System	Learning set and validation set of patients	Pretest/Posttest	Measures of diagnostic accuracy and intra & interobserver values	Developed	Not specified	2013	Australia
Can smartphone wireless ECGs be used to accurately assess ECG intervals in pediatrics? A comparison of mobile health monitoring to standard 12-lead ECG.	Gropler, M. et al.	Alivecor Kardia Mobile (KM)	Arrythmia	Cardiology	Monitoring	Wireless mobile health (mHealth) device	Smartphone	iPhone Operating System	Pediatric outpatients age <18 years presenting for cardiology clinic visits. 3 groups based on age: 0–5 years, 6–10 years, and 11–18 years	Experimental	Measures of diagnostic accuracy	Developed	Not specified	2018	United States
A Smartphone Application to Diagnose the Mechanism of Pediatric Supraventricular Tachycardia.	Ferdman, D., Liberman, L., Silver, E.	AliveCor monitor	Pediatric supraventricular tachycardia	Cardiology	Diagnosis	Smartphone application	Smartphone	iPhone Operating System	Pediatric patients undergoing an ablation for SVT	Experimental	Not specified	Developed	Not specified	2015	United States
A single center randomized, controlled trial investigating the efficacy of a mHealth ECG technology intervention to improve the detection of atrial fibrillation: the iHEART study protocol	Hickey, K.T. et al.	AliveCor® Mobile ECG	Atrial fibrillation	Cardiology	Diagnosis	Smartphone application	Smartphone	iPhone Operating System	Participants with a recent history of atrial fibrillation	Experimental	Not specified	Developed	Not specified	2016	United States
Excellent Symptom Rhythm Correlation in Patients with Palpitations Using A Novel Smartphone Based Event Recorder	William George Newhama, Muzahir Hassan Tayebjee	AliveCorECG	Palpitations	Cardiology	Diagnosis	Mobile application	Smartphone	Not specified	Patients with palpitations	Experimental	Not specified	Available	At £74.99 unit cost of the 2nd generation AliveCor monitor (2015)	2017	United Kingdom
Smartphone ECG for evaluation of STEMI: results of the ST LEUIS Pilot Study	Muhlestein, J.B. et al.	AliveCor™ Heart Monitor	STEMI	Cardiology	Monitoring	Smartphone application	iPod Touch 5th Gen	iPhone Operating System	Patients for whom the hospital STEMI protocol was activated	Pre-Posttest	Not specified	Developed	Not specified	2015	Multinational
Reliability and validity of a smartphone pulse rate application for the assessment of resting and elevated pulse rate	Mitchell, K. et al.	Azumio®'s Instant Heart Rate	Pulse rate	Cardiology	Monitoring	Smartphone application	Smartphone	iPhone Operating System and Android	"Adults from the Texas Woman's University School of Physical Therapy – Houston Campus"	Pre-Posttest	Not specified	Developed	Not specified	2016	United States
Testing feasibility of an accurate microscopic assessment of macrovesicular steatosis in liver allograft biopsies by smartphone add-on lenses.	Cesaretti, M. et al.	BLIPS Ultra lens	Macrovesicular steatosis (MS)	Clinical Lab	Diagnosis	Smartphone with lens adaptor	Smartphone	iPhone Operating System	Liver donors aged > 18 y who underwent surgical liver biopsy	Experimental	Correlation values	Available	Ultra Blips Lens: 10 Euro Together with stage and light source: about 25 Euro	2017	France

Assessment of a smartphone app (Capstesia) for measuring pulse pressure variation: agreement between two methods: A Cross-sectional study.	Barrachina, B. et al.	Capstesia	Postoperative complications	Hematology and Hemodynamics	Monitoring	Smartphone application	Smartphone	Android	Patients who had an arterial catheter (radial or femoral artery) inserted for haemodynamic monitoring	Observational	Measures of diagnostic accuracy and intra & interobserver values	Developed	Not specified	2017	Spain
Validation of a Standalone Smartphone Application for Measuring Heart Rate Using Imaging Photoplethysmography	Poh, M.Z. et al.	Cardiio	Pulse rate	Cardiology	Monitoring	Smartphone application	Smartphone	iPhone Operating System	Healthy adult volunteers	Experimental	Measures of diagnostic accuracy	Developed	Not specified	2016	United States
Diagnostic Performance of a Smartphone-Based Photoplethysmographic Application for Atrial Fibrillation Screening in a Primary Care Setting.	Chan, P. et al.	Cardiio Rhythm	Atrial fibrillation	Cardiology	Screening	Smartphone application	Smartphone	iPhone Operating System	Patients with hypertension, with diabetes mellitus, and/or aged ≥65 years	Pre-Posttest	Measures of diagnostic accuracy	Developed	Not specified	2016	Hong Kong
Contact-Free Screening of Atrial Fibrillation by a Smartphone Using Facial Pulsatile Photoplethysmographic Signals	Bryan P. Yan, et al	Cardiio Rhythm (Cardiio Inc, Cambridge, MA) smartphone application	Atrial fibrillation	Cardiology	Screening	Smartphone application	Smartphone	iPhone Operating System	Sample of 217 subjects	Experimental	Measures of diagnostic accuracy and correlation values	Developed	Not specified	2018	United States
Diagnostic Accuracy of a Novel Mobile Phone Application for the Detection and Monitoring of Atrial Fibrillation	Rozen, G. et al.	Cardiio Rhythm Mobile Application (CRMA)	Atrial fibrillation	Cardiology	Diagnosis/ Monitoring	Smartphone application	Smartphone	iPhone Operating System	Patients	Experimental	Measures of diagnostic accuracy	Developed	Not specified	2018	Multinational
Passive Detection of Atrial Fibrillation Using a Commercially Available Smartwatch	Tison, G.H. et al.	Cardiogram Inc	Atrial fibrillation	Cardiology	Diagnosis	Mobile application	Apple watches	iPhone Operating System	Health eHeart Study participants with an Apple Watch	Experimental	Measures of diagnostic accuracy	Developed	Not specified	2018	United States
Design and pilot results of a mobile phone weight-loss application for women starting a meal replacement programme	Brindal, E. et al.	Celebrity Slim MRP	Weight loss	Nutrition	Monitoring	Smartphone application	Smartphone	iPhone Operating System	Overweight or obese adult women	Experimental	Variability measures	Developed	Not specified	2013	Australia
Mobile microscopy as a screening tool for oral cancer in India: A pilot study	Skandarajah, A. et al.	CellScope	Oral cancer	Oncology	Diagnosis	Mobile phone-based microscope	Tablet	iPhone Operating System	Patients attending the dental out-patient departments of the Mazumdar-Shaw Medical Center (MSMC), Bangalore and its collaborative partner the KLE Society's Dental College (KLESDC), Bangalore.	Pre-Posttest	Measures of diagnostic accuracy	Developed	Not specified	2017	India
A Smartphone-Based Tool for Rapid, Portable, and Automated Wide-Field Retinal Imaging.	Kim, T. et al.	Cellscope Retina	Retinal diseases	Ophthalmology	Screening	Smartphone application	Smartphone	iPhone Operating System	Subjects from the University of Michigan Kellogg Eye Center Retina Clinic	Experimental	Measures of diagnostic accuracy	Developed	Not specified	2018	United States
Reliability Analysis of a Smartphone-aided Measurement Method for the Cobb Angle of ScoliPhone Operating Systemis	Qiao, J. et al.	Cobbmeter	Adolescent Idiopathic ScoliPhone Operating Systemis	Orthopedics and Traumatology	Diagnosis	Smartphone application	Smartphone	iPhone Operating System	Posteroanterior radiographs of adolescent idiopathic scoliPhone Operating Systemis patients with thoracic scoliPhone	Pre-Posttest	Intra & interobserver values	Developed	Not specified	2011	China

									Operating Systemis						
Clinical chemistry measurements with commercially available test slides on a smartphone platform: Colorimetric determination of glucose and urea	Yuanyuan Wu et al	ColorAssist	Diagnostics for urea and glucose	Clinical lab	Diagnosis	Smartphone application	Smartphone	iPhone Operating System	Canine whole blood samples were collected from five different dogs	Technical testing	Diverse measurements	Developed	Not specified	2015	United States
Hormonal Smartphone Diagnostics.	P. R. Matías-García, J. L. Martinez-Hurtado, A. Beckley, M. Schmidmayr, and V. Seifert-Klauss	Colorimetrix	Reproductive and metabolic disorders	Maternal and child health	Monitoring	Smartphone application	Smartphone	iPhone Operating System	None	Technical testing	None	Available	Not specified	2018	Multinational
Kidney Smartphone Diagnostics	P. R. Matías-Garcıa, J. L. Martinez-Hurtado	Colorimetrix	Kidney damage	Nephrology	Monitoring	Mobile application	Smartphone	iPhone Operating System	None	Technical testing	None	Available	Not specified	2018	Australia
Reliability of a smartphone-based goniometer for knee joint goniometry	Ferriero, G. et al.	DrGoniometer (DrG)	Knee joint goniometry	Orthopedics and Traumatology	Diagnosis	Smartphone application	Smartphone	iPhone Operating System	None	Technical testing	None	Developed	Not specified	2013	Italy
A Mobile Phone-Based Approach for Hearing Screening of School-Age Children: Cross-Sectional Validation Study.	Chu, Y. et al.	Ear Scale App	Hearing status	Otorhinolaryngology	Screening	Smartphone application	Smartphone and Tablet	iPhone Operating System	School-age children	Observational	Measures of diagnostic accuracy	Developed	Free	2019	Taiwan
Initial assessment of hearing loss using a mobile application for audiological evaluation	Derin, S. et al	Ear Trumpet app	Hearing loss	Otorhinolaryngology	Diagnosis	Mobile application	Smartphone	iPhone Operating System	Patients with hearing loss	Pre-Posttest	Not specified	Developed	Not specified	2016	Turkey
Automated audiometry using apple iPhone Operating System-based application technology.	Foulad, A., Bui, P., Djalilian, H.	EarTrumpet	Audiometry	Otorhinolaryngology	Diagnosis	Smartphone application	Smartphone, iPod and Tablet	iPhone Operating System	Subjects in the University of California, Irvine Medical Center neurotology clinic during their standard appointment for hearing evaluation	Experimental	Not specified	Developed	Not specified	2013	United States
Validity of the Elite HRV smartphone application for examining heart rate variability in a field-based setting	Perrotta, A.S. et al.	Elite HRV	Heart rate	Cardiology	Monitoring	Smartphone application	Smartphone	Not specified	Recreational athletes	Pre-Posttest	Measures of diagnostic accuracy and variability measures	Developed	Not specified	2017	Canada
Utility of the EncephalApp Stroop Test for covert hepatic encephalopathy screening in Chinese cirrhotic patients.	Xin Zeng Et al	EncephalApp	Hepatic encephalopathy	Gastroenterology	Screening	Smartphone and tablet app	Smartphone and tablet	Not specified	Cirrhotic patients	Experimental	Measures of diagnostic accuracy	Developed	Not specified	2019	China
Validation of EncephalApp, Smartphone-based Stroop Test, for the Diagnosis of Covert Hepatic Encephalopathy.	Bajaj, J. et al.	EncephalApp	Covert hepatic encephalopathy	Neurology	Diagnosis	Smartphone-based stroop test	Smartphone, iPod and tablet	iPhone Operating System	Patients with cirrhosis and controls	Experimental	Measures of diagnostic accuracy and intra & interobserver values	Developed	Not specified	2015	United States
Endoscope-i: an innovation in mobile endoscopic technology transforming the delivery of patient care in otolaryngology	N Mistry, C Coulson, A George	Endoscope-i	Patient care	Otorhinolaryngology	Diagnosis/ Screening	Endoscopic Mobile Image System	Smartphone	iPhone Operating System	None	Technical testing	None	Developed	£ 999.00 (aprox)	2017	United Kingdom
Diabetes self-management smartphone application for adults	Kirwan, M. et al.	Glucose Buddy	Diabetes	Endocrinology	Monitoring	Smartphone application	Smartphone	iPhone Operating System	Adult patients with type 1 diabetes	Experimental	Variability measures	Developed	Free	2013	Australia

with type 1 diabetes: randomized controlled trial															
Pocket mobile smartphone system for the point-of-care submandibular ultrasonography	Wojtczak, J., & Bonadonna, P.	Mobisante MobiUS system	Airway	Anesthesiolo gy	Diagnosis	Smartphone-based application	Toshiba smartphon e	Not specified	Healthy (body mass index <30) volunteers	Experimental	Variability measures	Developed	Not specified	2013	United States
